# Genetic and Biochemical Investigation of Seed Fatty Acid Accumulation in Arabidopsis

**DOI:** 10.3389/fpls.2022.942054

**Published:** 2022-07-06

**Authors:** Chinedu Charles Nwafor, Delin Li, Ping Qin, Long Li, Wei Zhang, Yuanwei Zhou, Jingjing Xu, Yongtai Yin, Jianbo Cao, Limin He, Fu Xiang, Chao Liu, Liang Guo, Yongming Zhou, Edgar B. Cahoon, Chunyu Zhang

**Affiliations:** ^1^National Key Laboratory of Crop Genetic Improvement, College of Plant Science and Technology, Huazhong Agricultural University, Wuhan, China; ^2^Department of Crop Science, Faculty of Agriculture, University of Benin, Benin City, Nigeria; ^3^Department of Biology, College of Chemistry and Life Sciences, Zhejiang Normal University, Jinhua, China; ^4^Yichang Academy of Agricultural Science, Yichang, China; ^5^Department of Biotechnology, College of Life Science and Technology, Huazhong University of Science and Technology, Wuhan, China; ^6^Public Laboratory of Electron Microscopy, Huazhong Agricultural University, Wuhan, China; ^7^Collaborative Innovation Center for the Characteristic Resources Exploitation of Dabie Mountains and College of Biology and Agriculture Resource, Huanggang Normal University, Huanggang, China; ^8^Center for Plant Science Innovation and Department of Biochemistry, University of Nebraska-Lincoln, Lincoln, NE, United States

**Keywords:** fatty acid biosynthesis, chlorophyll synthase, photosynthesis, oxidative pentose phosphate pathway, NADPH, oil bodies, Arabidopsis, seed development

## Abstract

As a vegetable oil, consisting principally of triacylglycerols, is the major storage form of photosynthetically-fixed carbon in oilseeds which are of significant agricultural and industrial value. Photosynthesis in chlorophyll-containing green seeds, along with photosynthesis in leaves and other green organs, generates ATP and reductant (NADPH and NADH) needed for seed fatty acid production. However, contribution of seed photosynthesis to fatty acid accumulation in seeds have not been well-defined. Here, we report the contribution of seed-photosynthesis to fatty acid production by probing segregating green (photosynthetically-competent) and non-green or yellow (photosynthetically-non-competent) seeds in siliques of an Arabidopsis chlorophyll synthase mutant. Using this mutant, we found that yellow seeds lacking photosynthetic capacity reached 80% of amounts of oil in green seeds at maturity. Combining this with studies using shaded siliques, we determined that seed-photosynthesis accounts for 20% and silique and leaf/stem photosynthesis each account for ~40% of the ATP and reductant for seed oil production. Transmission electron microscopy (TEM) and pyridine nucleotides and ATP analyses revealed that seed photosynthesis provides ATP and reductant for oil production mostly during early development, as evidenced by delayed oil accumulation in non-green seeds. Transcriptomic analyses suggests that the oxidative pentose phosphate pathway could be the source of carbon, energy and reductants required for fatty acid synthesis beyond the early stages of seed development.

## Introduction

Seeds accumulate oil in the form triacylglycerol (TAG) as one of their primary storage reserves. TAG is a major energy source for germinating seeds and for human and animal diets. Seed oil content is a major target of breeding and biotechnological efforts to increase the value of oilseed crops such as soybean and canola for edible, industrial, and biofuel applications (Cahoon et al., 2007; Lu et al., 2011). Oil production in seeds starts with *de novo* fatty acid (FA) biosynthesis and elongation, followed by the use of these fatty acids for TAG biosynthesis (Baud and Lepiniec, 2009; Hua et al., 2014). Acetyl-CoA production and the generation of reducing power from NADPH and energy from ATP are essential for FA biosynthesis, elongation, and storage (Singh et al., 1998; Ruuska et al., 2004; Goffman et al., 2005; Zhang et al., 2016).

Chlorophyll is important for light-driven production of reductants and energy that supports growth and development and formation of seed storage components, including seed oil (Ruuska et al., 2004; Schwender et al., 2004; Goffman et al., 2005; Hua et al., 2014; Zhang et al., 2016). Developing seeds can be classified as either photoheterotrophic green seeds (e.g., soybean, rapeseed, and Arabidopsis) or heterotrophic non-green seeds (e.g., castor, sunflower, and safflower; Zhang et al., 2016). The biochemical pathways and their subcellular distribution for FA biosynthesis in seeds are well-understood (Ohlrogge and Browse, 1995; Eastmond et al., 1996; Cernac and Benning, 2004; Allen et al., 2009; Li-Beisson et al., 2010; Troncoso-Ponce et al., 2011; Bates et al., 2013; Shahid et al., 2019). Less clear are the major tissue and organ sources of reductant for fatty acid biosynthesis in green seeds. Does reductant derive primarily from photosynthesis in leaves and stems, seed pods and siliques, or seeds or from metabolism of imported carbon in these tissues? Previous studies that investigated the role of light and seed photosynthesis in rapeseed (Fuhrmann et al., 1994; Willms et al., 1999), Arabidopsis (Goffman et al., 2005), and soybean (Quebedeaux and Chollet, 1975; Ruuska et al., 2004; Allen et al., 2009; Tschiersch et al., 2011), have proposed several hypotheses on the sources of reducing power for oil biosynthesis and accumulation (Fuhrmann et al., 1994; Ruuska et al., 2004; Schwender et al., 2004; Goffman et al., 2005). So far, it is believed that fatty acid synthesis and oil accumulation in green seeds, such as soybean is strongly controlled by light which is necessary for grain filling (Allen et al., 2009), and light-dependent seed photosynthesis is the major source of reductive power supporting FA biosynthesis and elongation in green seeds (Fuhrmann et al., 1994; Goffman et al., 2005).

Multiple labeling experiments (Fuhrmann et al., 1994; Goffman et al., 2005; Rolletschek et al., 2005; Allen et al., 2009; Lonien and Schwender, 2009; Tschiersch et al., 2011; Carey et al., 2020), have described energy balances and biosynthetic flux in growing embryos *in vitro*, and have provided insights into photosynthetically-derived energy and carbon flux required for seed oil production. Because these studies were conducted with excised embryos exposed to light, the findings may not fully mimic *in situ* metabolic flux that can be obtained by assessment of metabolic flux in seeds attached to plants, because dissecting plant tissues is known to impair metabolism (Geigenberger et al., 1994; Ruuska et al., 2004).

A growing body of evidence has brought into question the contribution of seed photosynthesis to reducing power required for fatty acid and oil biosynthesis in seeds (Borisjuk et al., 2005; Borisjuk and Rolletschek, 2009). One report that argues against the role of seed photosynthesis, suggests that G6PDH plays a role in supplying NADPH for oil accumulation in developing seeds in which photosynthesis may be light limited (Wakao et al., 2008). The authors showed that (pds1) mutants seeds that are non-photosynthetic had 60% of oil in photosynthetic green seeds at maturity and concluded that loss of cytosolic G6PDH activity impacted metabolism of developing seeds by increasing carbon substrates for synthesis of storage compounds rather than by decreasing the NADPH supply specifically for fatty acid synthesis. Additionally, Eastmond et al. (1996) suggests that the low light penetrance through *Brassica napus* siliques is insufficient for photosynthetic reductant production in seeds and that reductant supply to the plastid may underpin storage product synthesis. Also, it has been shown that loss of photosynthetic capacity in the soybean embryo is accompanied by a steady accumulation of starch and lipids bodies (Borisjuk et al., 2005). Together these results points to perhaps limited metabolic contribution of seed photosynthesis to fatty acid production in seeds during development.

In this report, we aimed to provide genetic evidence on the contribution of seed-photosynthesis to fatty acid biosynthesis during seeds development. To which we exploited the Arabidopsis chlorophyll synthase knockout mutant and its complement that segregate green (photosynthetically-competent) and, non-green or yellow (photosynthetically-non-competent) seeds in the silique combined with electron microscopy, and chlorophyll and fatty acid measurements, RNA-seq based transcriptomics/gene expression analysis, and LC/MS/MS-based Pyridine nucleotides and ATP analysis. Our results showed that photosynthesis in seeds has only a small contribution to NADPH reductant needed for fatty acid synthesis and deposition. The LC–MS/MS and gene expression studies provided clues that supported a role for the oxidative pentose phosphate pathway (OPPP) as a major pathway for NADPH production for fatty acid synthesis in Arabidopsis seeds. Also, we highlighted the metabolic impact of lacking chlorophyl in developing seeds and the potential biotechnological implication of metabolic engineering of both photosynthetic and OPPP pathway genes for enhancing seed oil production through efficient NADPH supply in developing seeds.

## Materials and Methods

### Plant Material and Growth Conditions

The *Arabidopsis thaliana* Wild-type Col-0 ecotype, chlorophyll synthase knockout mutant (chlsyn1-1) and complemented (chlsyn1-1/CHLSYN) lines used for this study was previously described by Zhang et al. (2015). All plants were grown in the glasshouse at 22°C with 50% relative humidity and 16 h light (120 μE m^−2^ s^−1^/8 h dark).

### Sampling of Plant Material

Developing silique were labeled throughout the flowering period (Lorenz et al., 2018). Siliques produced between the third and seventh flower on the main axis were used. For tagging of each stage of seed development, we classified green and yellow seed developing seeds into (6, 8, 10, 12, 14, 16, and 18 days after flowering (DAF), and dry seeds at 40 DAF), two or three experimental replicates were achieved. For the shading experiment of siliques from light penetration, aluminum foil was used to cover siliques for the whole life, and unshaded siliques were used as control. The foils were draped around the siliques and with vents at the tip to allow air circulation. For FAs analysis, at least 48 plants of each of the WT and mutant lines were used. For histological studies, seeds from the same silique were used (at least three siliques from each line). For RNA-seq and LC–MS/MS-based pyridine nucleotides and ATP analysis, seeds were collected from more than 96 plants for each replicate and developmental stage. All plants were grown side by side under identical conditions. The photosynthetic, green seeds were used as control.

### Measurement of Chlorophyll Content

For estimation of the Total chlorophyll content was measured according to the method previously described (Zhang et al., 2015). Chlorophyll was extracted after freshly harvested seed were weighed and homogenized in liquid nitrogen, subsequently extracted in three volumes of 80% (v/v) acetone containing 1 μM KOH. After centrifugation for 2 min at 16,000 *g*, the supernatant was used for spectrophotometric analysis of chlorophyll concentrations.

### Fatty Acid Analysis and Protein Analysis

Fatty acid methyl esters (FAMEs) were prepared from developing seeds and dry seeds, as described in Niewiadomski et al. (2005). FAs estimation was performed by gas chromatography with flame ionization detector (GC-FID) after direct transesterification was made by the addition of 2.5% sulfuric acid in methanol (v/v) and incubating at 90°C for 2 h as described in Niewiadomski et al. (2005) and Cahoon et al. (2006). Fatty acid amounts were determined by comparing detector response relative to an internal standard (C17:0) from triheptadecanoin added prior to sample transesterification. Fatty acid composition was calculated as percentage of seed weight.

Protein content estimation was performed by extracting total protein from dry seeds as described in Job et al. (2005). In brief, seeds were homogenized to a fine powder in liquid nitrogen and then transferred to a 1.5 ml Eppendorf tube. Next, 10% TCA/0.07% dithiothreitol (DTT) in acetone (v/v), was added to the samples and incubated at −20°C overnight, followed by centrifugation for 30 min at 20,000 g at 4°C. Afterward, the pellets were then washed three times with 1.5 ml of pre-cooled acetone containing 0.07% DTT at −20°C followed by centrifugation for 30 min at 20,000 g at 4°C. The sample pellets were then solubilized in the lysis buffer containing 7 mM urea, 2 mM thiourea, 4% CHAPS, 50 mM DTT, 0.5% Triton X-100, 1% protease inhibitor cocktail, and 2% IPG buffer (v/v). The solution was incubated at 25°C for 1 h with gentle mixing and then centrifuged at 12,000 g for 20 min. The supernatant was subjected to second clarifying centrifugation as above with the supernatants collected into fresh tubes and stored at −80°C in aliquots. The spectrophotometer was used to quantify the proteins. Bovine serum albumin was used as a standard.

### Transmission Electron Microscopy

Transmission electron microscopy (TEM) was performed as described (Gan et al., 2013). For TEM, developing (8 and 12 DAF, and dry seeds 40 DAF), described in section Sampling of Plant Material were used. Developing and dry seeds from the same siliques were removed, cut into 1 mm × 1 mm blocks and fixed immediately in 2.5% (w/v) glutaraldehyde in 0.1 mol L–1 phosphate buffer solution (PBS) at 4oC overnight. The fixed samples were then rinsed with PBS three times for 30 min each at room temperature (20°C–25°C), and post-fixed in 1% OsO4 in phosphate buffer overnight at 4oC. Next, samples were washed with Milli-Qwater and dehydrated through an acetone series (20%, 50%, 70%, and 90%, and 3% × 100% v/v). The samples were cut into ultrathin sections (60–70 nm thick) using a diatome diamond knife on a UC6 Ultratome (Leica, Germany), stained with 2% uranyl acetate (v/v). The mages were viewed and collected with a Hitachi transmission electron microscope (TEM; H-7650; Hitachi, Japan) at 80 kv. Each sample had three biological replicates with each replicate having at least three ultrathin sections observed under the electron microscope. The imageJ software (Girish and Vijayalakshmi, 2004) was used to measure the area of oil bodies in embryos cotyledons from three sections of each of the three independent biological replicates of each sample.

### Pyridine Nucleotides and ATP Analyses by LC-MS/MS

Measurement of pyridine nucleotides and ATP analysis *via* LC-MS/MS was conducted as described previously in Guo et al. (2014). About 100 mg of green and yellow seeds at 8 and 10 DAFs were used. After the seed was harvested on ice and immediately ground in liquid nitrogen, 3 ml of methanol/chloroform (7:3, v/v; −20°C) was added to each sample, then mixed by vortex. Next, 0.9 μg PIPES was added as the internal standard, and the mixture was stored in −20° C for 2 h with occasional mixing. Subsequently, polar metabolites were extracted by the addition of 1.6 ml water followed by vigorous vortexing and centrifugation at 8,000 rpm for 20 min. Followed by the transfer of the upper methanol–water phase to a new tube. This step was repeated twice. Afterward, the first and second upper methanol–water phases were pooled and dried by N_2_ aspiration at room temperature. The dried extract was reconstituted with 100 μl water and filtered with 0.45 μM cellulose acetate centrifuge tube filter and then diluted by 10- or 100-fold for analyses by LC–MS/MS.

Absolute quantification of pyridine nucleotides and ATP metabolites (ATP, ADP, NAD, NADH, NADP, and NADPH), was achieved by the use of high purity external standards. For each metabolite, a calibration curve was made by preparing eight standard solutions with known concentrations in water. Each standard solution was analyzed three times and metabolites were determined by plotting average peak areas against standard concentration using a linear regression model.

### RNA-Seq Analysis

Total RNA from developing seeds was extracted with the TRIzol Reagent Kit (Ambion) according to the manufacturer’s protocol. The RNA quality and quantity were determined using a Nanodrop 8000 (Thermo Scientific, Wilmington, DE) and a Bioanalyzer 2100 (Agilent, Santa Clara, CA). Before RNA extraction, green and yellow developing seed at 10 DAF were separated from silique and quickly grounded in liquid nitrogen. All samples were collected in three biological replicates. Afterward, samples from each biological replicate were pooled. In total, six samples were used to construct the cDNA library with Illumina® TruSeq™ RNA Sample Preparation Kit following the manufacturer’s instructions. All samples were sequenced using an Illumina HiSeq 2000 sequencer at the Beijing Genome Institute, Shenzhen, China. Analysis of sequence data was according to the method described in Ramakers et al. (2003). RNA-seq sequence data can be found in the GEO database with ID no GSE152614.

### Real-Time Polymerase Chain Reaction

For validation of RNA-Seq data and expression analysis of other genes, quantitative real-time PCR (qRT-PCR) was performed on RNA obtained from all the biological replicates belonging to green and yellow seeds used for RNA sequencing. For all RNA samples, the first-strand cDNA was synthesized using the Thermo Scientific RevertAid Kit according to the manufacturer’s protocol. qRT-PCRs were completed with the SYBR Green Premix system (Newbio Industry) and gene-specific primers using the CFX Connect™ real-time PCR detection system (BIO-RAD, Hercules, CA). The expression profiles of all genes were analyzed, with Actin7 (AT5G09810) as the constitutive gene for normalization. PCR conditions were as follows 95°C for 1 min, for 44 cycles at 95°C, 12 s, 60°C, 30 s and 72°C, 30 s. After cycling, the melting curves of the reaction were run from 55°C to 95°C. Relative expression was calculated with the software LINREG, as described by Ling et al. (2017).

### Statistical Analysis

Each experiment was performed in triplicate except otherwise stated. All data are expressed as the means ± SDs. A Student’s *t*-test was used to interpret the differences among the data. Differences between groups were considered to be statistically significant at *p* ≤ 0.05.

## Results and Discussion

### Oil Concentrations Are Moderately Reduced in Mature Seeds Lacking Photosynthetic Capacity

**Figure 1:**
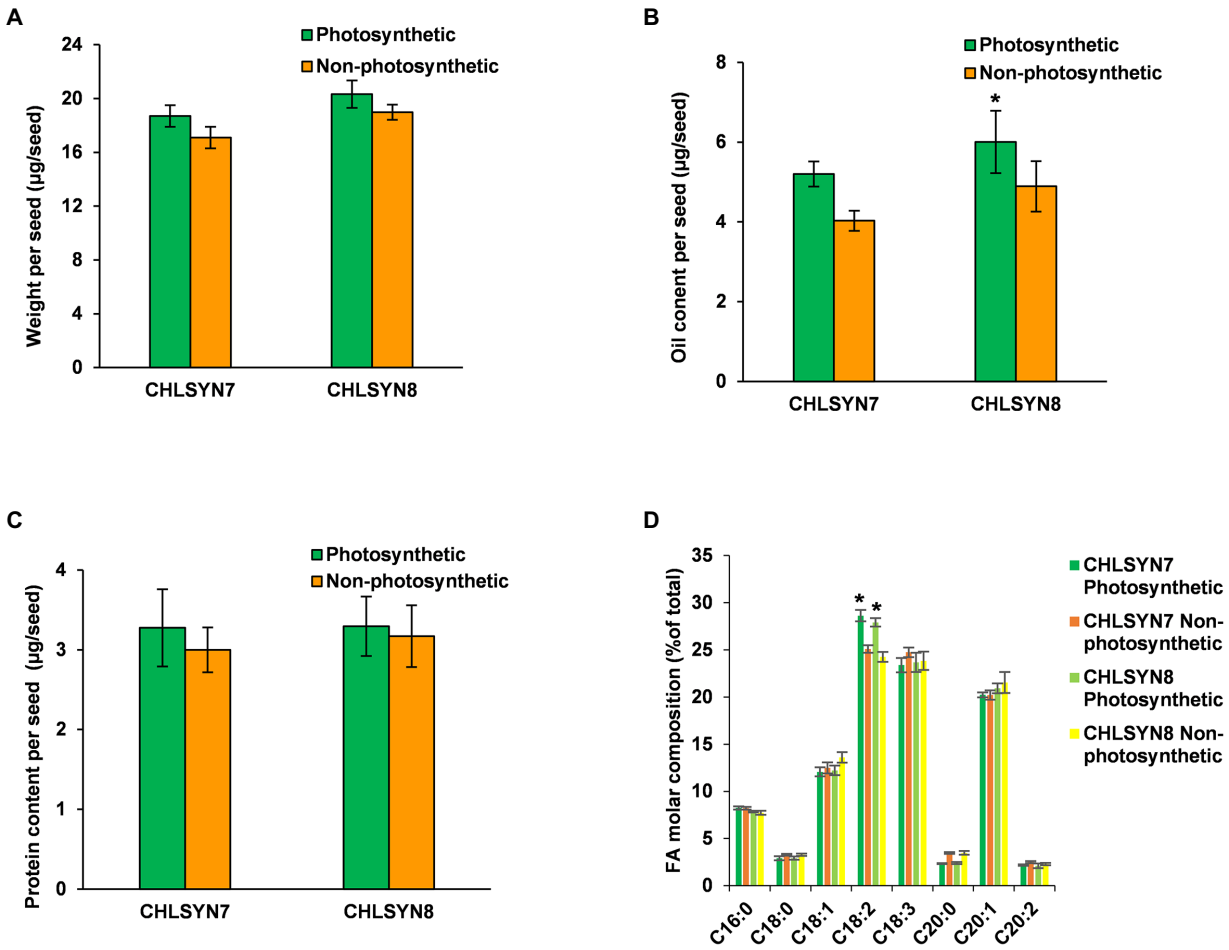
Analyses of seed biomass, oil, and protein contents. **(A)** Comparison of seed weight in photosynthetic, green seeds (red) and non-photosynthetic, yellow seeds (non-red) from two (CHLSYN7 and CHLSYN8) independent Atchlsyn1-1/CHLSYN lines. **(B)** FAMEs analysis of seed oil content of red/green (photosynthetic) seeds and, non-red/yellow (non-photosynthetic) seeds from two independent mutant lines CHLSYN7 and CHLSYN8, respectively. **(C)** Comparison of red/green (photosynthetic) seeds and, non-red/yellow (non-photosynthetic) seeds protein content in two independent complemented lines, CHLSYN7 and CHLSYN8. The two independent lines are represented by orange and green bars, respectively. **(D)** Fatty acid composition of seed wildtype and mutant seeds. Orange and cyan bar indicates green (photosynthetic) and yellow (non-photosynthetic) seeds of CHLSYN7, yellow and light-green bar indicates green (photosynthetic) and yellow (non-photosynthetic) seeds from CHLSYN8, respectively. Values are means ± SD (*n* = 3 biological replicates). Statistical significance was analyzed using the two-sided Student’s *t*-test. The asterisk indicates a significant difference (^*^p < 0.05).

In this study, we used the Arabidopsis chlorophyll synthase knockout mutant Atchlsyn1-1 complemented with the Arabidopsis chlorophyll synthase cDNA linked to a DsRed fluorescent protein marker (Zhang et al., 2015; Supplementary Figures S1A,B). This system allowed us to distinguish seeds in individual siliques segregating for lack of chlorophyll (non-photosynthetic) from those with chlorophyll (photosynthetic), which also fluoresced red from the DsRed marker. This system was advantageous for our studies because photosynthetic and non-photosynthetic seeds could be readily identified at the very earliest stages of seed development and dry seeds. For these experiments, we used two independent complemented lines of Atchlsyn1-1 designated “CHLSYN7” and “CHLSYN8” (Supplementary Figures S1A–C). To understand the effects of loss of photosynthetic capacity, we measured seed biomass, oil and protein contents, and fatty acid composition in non-photosynthetic and photosynthetic dry seeds (40 DAF). Seed weight was reduced by ~8% in non-photosynthetic (non-red) seeds compared to the complemented photosynthetic (red) seeds (Figure 1A). FA amount in seeds were significantly reduced by ~20% (Figure 1B) and protein amount in seeds were ~6% lower in non-photosynthetic seeds relative to photosynthetic seeds (Figure 1C). The only significant difference in fatty acid composition between these seeds was a reduction in linoleic acid content from 29% of total fatty acids in photosynthetic seeds to 25% in non-photosynthetic seeds (Figure 1D). We suspect the reduction in C18:2 could result from lack of photosynthesis which could limit the lengthening of fatty acid chain in the non-photosynthetic seeds during development. Overall, non-photosynthetic seeds accumulated ~80% of oil relative to photosynthetic seeds at maturity.

### Non-photosynthetic Seeds Display Significantly Reduced Oil Content During Early Seed Development

**Figure 2:**
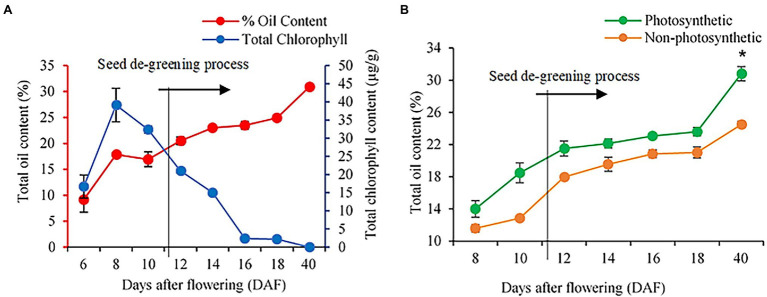
Analysis of seed oil and chlorophyll content in WT and Atchlsyn1-1 mutant during development. **(A)** Biochemical estimation of total FAs and chlorophyll content in WT. Oil content is on the left or the primary Y-axis and chlorophyll content is on the right or secondary Y-axis. **(B)** The seed oil content of seeds from photosynthetic, green seeds (red), and non-photosynthetic, yellow (non-red) seeds. Blue lines indicate WT seed chlorophyll content. The Red line indicates WT seed oil content. The green line indicates photosynthetic, green/(red) seed oil content. The orange line indicates non-photosynthetic, yellow/(non-red) seed oil content. Values are means ±SD (*n* = 3 biological replicates). Statistical significance was analyzed using the two-sided Student’s *t*-test. The asterisk indicates a significant difference (^*^p < 0.05).

In many photosynthetic organisms, previous studies have linked photosynthesis activity to chlorophyll concentrations (De La Harpe et al., 1979; Kura-Hotta et al., 1987; Gratani et al., 1998; Lodish et al., 2000; Baker and Oxborough, 2004; Govindjee, 2004; Raven et al., 2005), These studies indicated that chlorophyll is essential for photosynthesis because of the porphyrin ring of Chl-a and b that interacts directly in the light requiring reactions of photosynthesis. In the present study, we found that the non-photosynthetic seeds at early developmental stages had no residual chlorophyll pigment and chlorophyll levels were also not detectable in these seeds by our method (see Supplementary Figure S1; Zhang et al., 2015). We next explored the stages of seed development that are most affected in seed oil accumulation in the non-photosynthetic seeds. For these studies, we measured oil and chlorophyll content in seeds over a time course of 6 DAF to maturity (Figure 2). In wild-type seeds, oil content increased over seed development, while chlorophyll concentrations were highest at 8 DAF and declined gradually at 16 DAF to <5% of 8 DAF concentrations (Figure 2A). Of note, no significant differences in chlorophyll content were observed during development in seeds of complemented Atchlsyn1-1 plants compared to those of wild-type plants (Supplementary Figure S2). Seeds lacking chlorophyll had significantly lower oil content than that of wild-type seeds at 8 DAF, but oil content increased to 80% of wild-type oil content by 40 DAF (Figure 2B).

**Figure 3:**
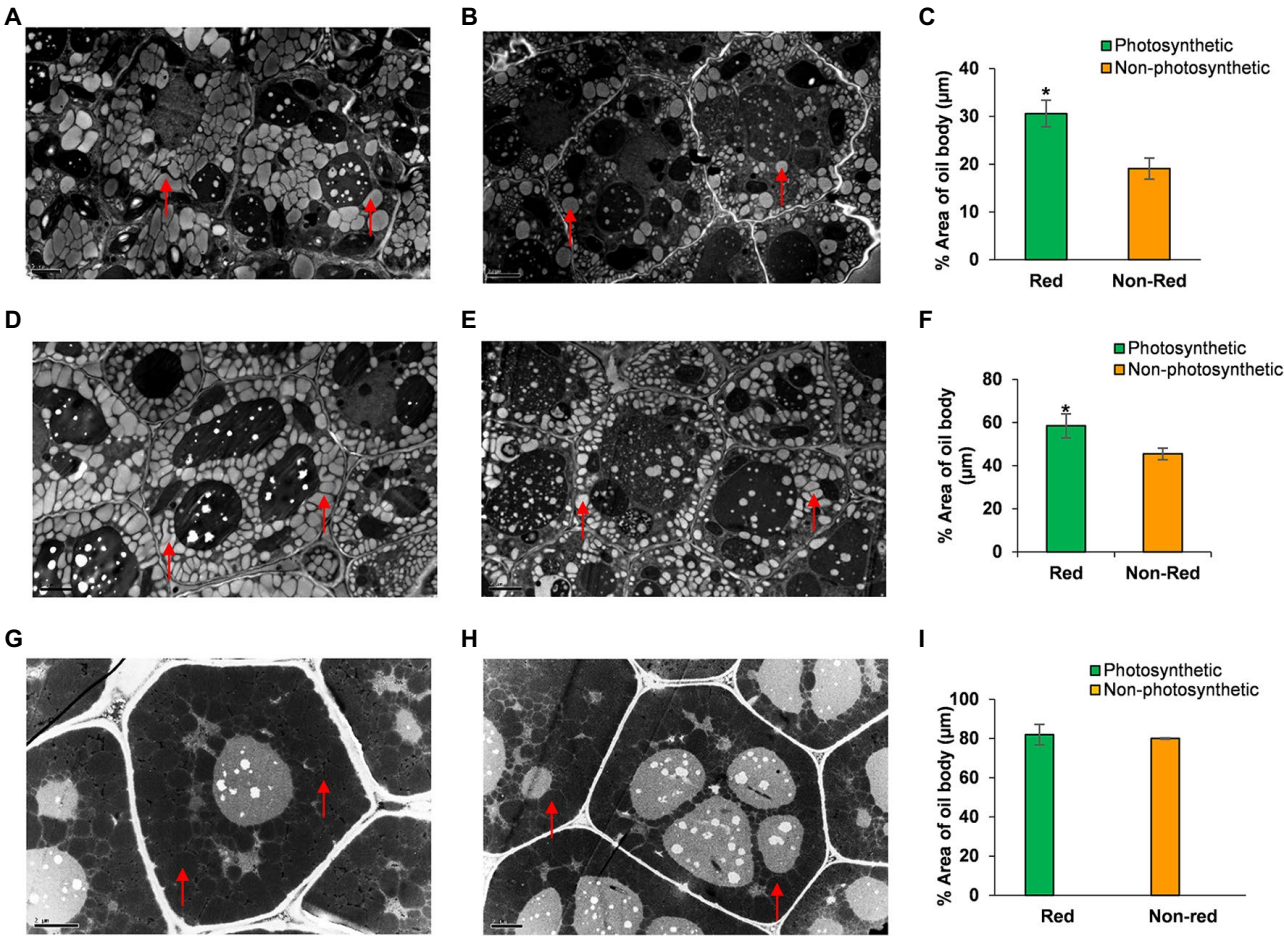
TEM analysis of oil bodies of embryos cotyledons from the same silique of Arabidopsis chlorophyll synthase mutant line CHLSYN7. **(A)** Section of developing embryos from photosynthetic, green(red) seeds at 8 DAF. **(B)** Section of developing embryos from non-photosynthetic, yellow(non-red) seeds at 8 DAF. **(C)** Percentage area of oil bodies per cell of embryos from photosynthetic, green/(red) and non-photosynthetic yellow(non-red) seeds at 8 DAF. **(D)** Section of developing embryos from photosynthetic, green(red) at 12 DAF. **(E)** Section of developing embryos from non-photosynthetic, yellow(non-red) seeds at 12 DAF. **(F)** Percentage area of oil bodies per cell of embryos cotyledons from photosynthetic, green/(Red) and non-photosynthetic, yellow (Non-red) seeds at 12 DAF. **(G)** Section of embryos from dry seeds (Red). **(H)** Section of embryos from dry non-red seeds. **(I)** Percentage area of oil bodies per cell of embryos cotyledons from dry seeds (40 DAF) red and non-red. Red triangles indicate the oil body. Green and orange bars indicate red and non-red seeds of CHLSYN7 seeds. Scale bar = 2 μm. Values are means from 5 to 6 section obtained from three replicates. Statistical significance was analyzed using the two-sided Student *t*-test. The asterisk indicates a significant difference (^*^p < 0.05).

To better understand how seed photosynthesis affects oil accumulation during development, we examined the ultrastructural characteristics of oil bodies of developing (8 and 12 DAF) and dry seeds (40 DAF) from non-photosynthetic, yellow/non-red and photosynthetic, and green/red seeds at different stages of development (Figure 3; Supplementary Figure S3). TEM analysis showed that the developing embryos cotyledons from non-photosynthetic yellow/non-red seeds had fewer and small oil bodies compared to the embryos cotyledons from the photosynthetic, green seeds at the early stages of 8 and 12 DAF (Figures 3A–F). Also, the percentage area covered by oil bodies was significantly lower (*p* ≤ 0.05) in the non-photosynthetic, yellow sheds when compared to the photosynthetic, green seeds (Figures 3C,F). Remarkably, there were no observable differences in the oil bodies of embryos cotyledons from photosynthetic and non-photosynthetic dry seeds (Figures 3G–I), the percentage area of the oil-bodies (dark regions of g and h) observed in embryos in these seeds at maturity was not significantly different (*p* > 0.05; Figure 3I).

Overall, these findings indicate that lack of chlorophyll which inhibited seed-photosynthesis in these Arabidopsis seeds largely affects oil accumulation during early development, and another sources of reductant supply largely support these requirements for oil production during subsequent developmental stages both in yellow and green seeds.

### Quantification of Photosynthetic Organs Contribution to Total Oil Production in Arabidopsis Seeds

**Figure 4:**
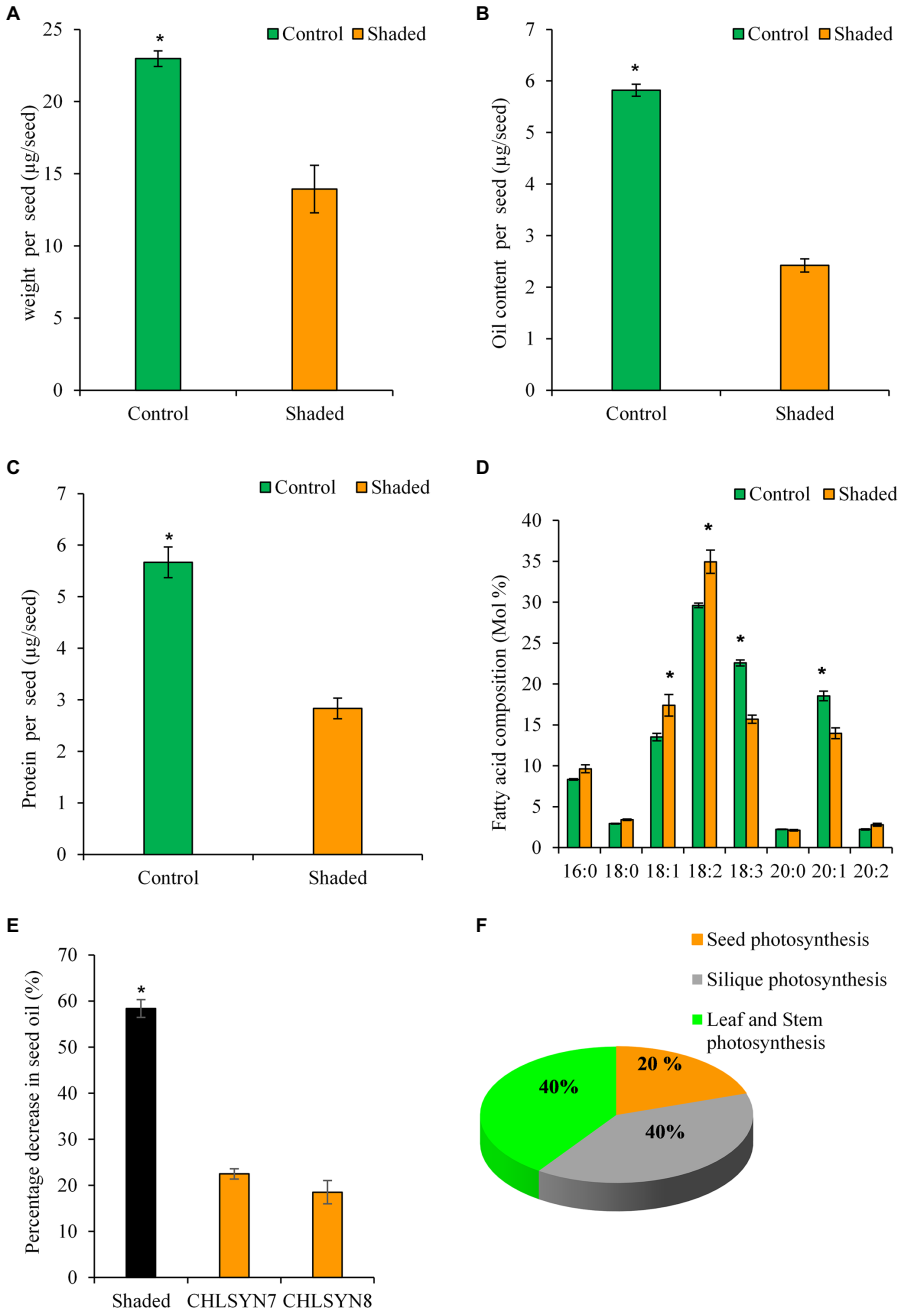
Photosynthetic organ contribution to total oil production in seeds. **(A)** Analysis of the weight of dry shaded and control seeds (40 DAF). **(B)** The total oil content of dry shaded and control seeds. **(C)** The protein content of dry shaded and control seeds. **(D)** Fatty acid composition of shaded and control seeds. **(E)** The relative decrease in oil content per plant. **(F)** Contribution of different photosynthetic tissues to seed oil production (seed chlorophyll ± error = 1.53, silique chlorophyll ± error = 1.97). Values are means ± SD (*n* = 3 biological replicates). Green and orange bars indicate control and shaded. Statistical significance was analyzed using the two-sided Student’s *t*-test. The asterisk indicates a significant difference (^*^p < 0.05).

In this study, we have shown that seed photosynthesis is important for oil deposition during early development as seen in the delay of oil accumulation (Figure 2) and in total accounts for ~20% of total FA for Arabidopsis seeds (Figure 1). To examine the organ photosynthetic source for the remaining 80% of FA in Arabidopsis seeds, we shaded siliques of wild-type Arabidopsis over their entire development to completely block light penetration (Supplementary Figure S4). Dry seeds from shaded siliques had a ~40% reduction in weight relative to those from unshaded control siliques (Figure 4A). Seed oil content was reduced by ~59% (Figure 4B) and protein content was decreased by ~50% (Figure 4C) in seeds from shaded siliques compared to those from unshaded siliques. The most significant changes in fatty acid composition in seeds from shaded siliques versus unshaded siliques were an increase in oleic acid (18:1) and linoleic acid (18:2) balance by decrease in relative amounts of linolenic acid (18:3) and eicosenoic acid (20:1; Figure 4D). Here, it is likely that shading could lead to low oxygen levels in the silique which may contribute to the significant reduction observed in seed weight and oil content. Also, blocking photosynthesis through shading in developing seeds, could limit carbon, cofactors, and reductants supply required for *de novo* fatty acid biosynthesis and possibly inhibited elongation of fatty acid chains in the shaded seeds resulting in variation in the proportions of fatty acids in shaded compared to unshaded. Nevertheless, by combining these results with those from Atchlsyn1-1 plants, we derived that silique photosynthesis contributes to ~40% of the seed oil biosynthesis and storage versus ~20% from seed photosynthesis (Figure 4E). By inference, leaf and stem photosynthesis contribute to the remaining 40% of seed oil production and accumulation (Figure 4F). Considering that seeds are attached to siliques, we conclude that photosynthetic products coming from the silique seems to be the most important contributor to seed oil production.

### Comparative Analysis of Pyridine Nucleotides and ATP Levels of Photosynthetic and Non-photosynthetic Seeds

**Figure 5:**
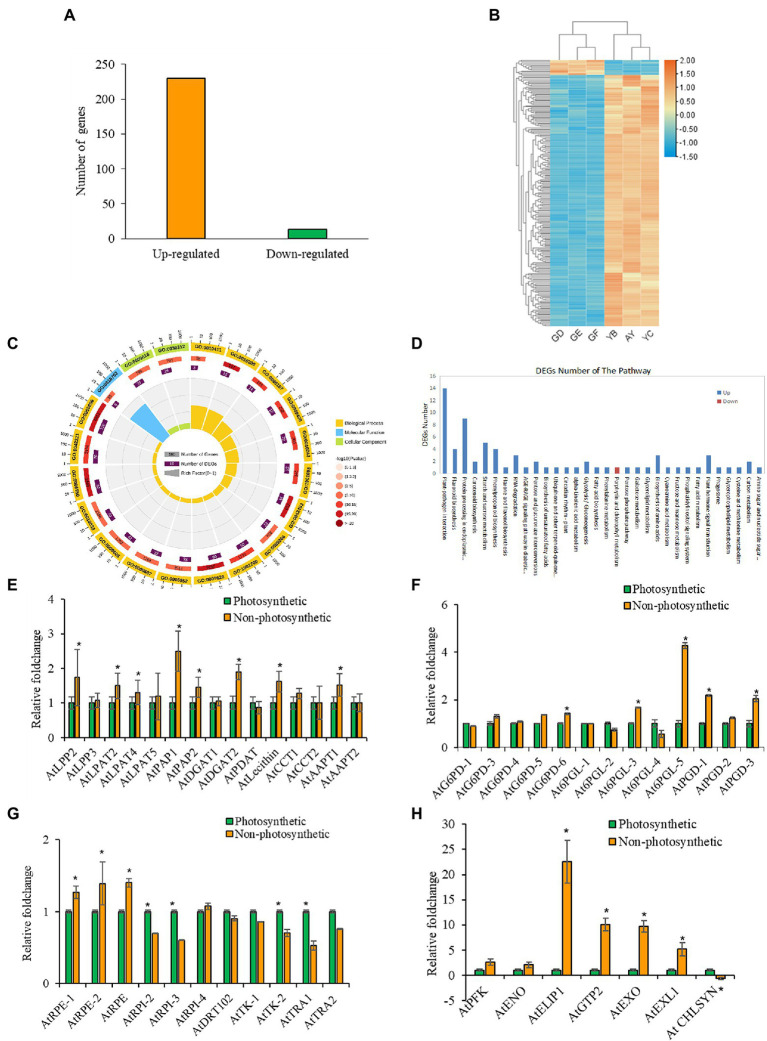
Gene expression analysis of developing yellow and green seeds at 10 DAF. **(A)** Number of DEGs in the non-photosynthetic background. **(B)** Heat map showing expression profile of DEGs. **(C)** Showing the GO classification of DEGs in the non-photosynthetic background. **(D)** Differentially enrich pathways of DEGs in the non-photosynthetic background. **(E)** Real-time PCR analysis of TAG biosynthetic pathway genes. **(F)** Differentially expressed gene of the cytosolic and plastic branch of the oxidative pentose phosphate pathway. **(G)** Non-oxidative pentose phosphate pathway. **(H)** Sugar transport. Green and yellow bars indicate photosynthetic green seeds and non-photosynthetic seeds. Values are means ±SD (*n* = 3 biological replicates, i.e., GD, GE, GF are three replicated biological samples belonging to green seeds. While YB, YA, YC are three replicated biological samples of yellow seeds). Statistical significance was analyzed using the two-sided Student *t*-test. The asterisk indicates a significant difference (^*^p < 0.05).

Cellular energy and reductant supply play a significant role in the overall plant physiology, including the production of seed storage reserves (Agrawal and Calvin, 1971; Wakao et al., 2008; Chen and Shachar-Hill, 2012; Carey et al., 2020). Photosynthesis provides both carbon source and reducing power directly for FA synthesis, and we have shown that seed-photosynthetic reductant supply is critical for seed oil production particularly at the early stages of development (Figures 2, 4). We next aimed to identify reducing power sources in seeds where photosynthetic capacity is absent and to clarify the metabolic consequence of lack of seed-photosynthesis-derived reducing power to seeds oil content during early stages of seed development. For these studies, we performed targeted LC–MS/MS-based quantification of the pool sizes of cofactors associated with fatty acid biosynthesis and storage and central metabolism in photosynthetic and non-photosynthetic seeds at 8 and 10 DAF (see Materials and Methods section, Figure 5; Supplementary Table S1). ATP, ADP, NADH, NAD, NADP, and NADPH levels were quantitatively determined, and their ratios estimated as a parameter for measuring seed cellular energy status (Figure 5).

**Figure 6:**
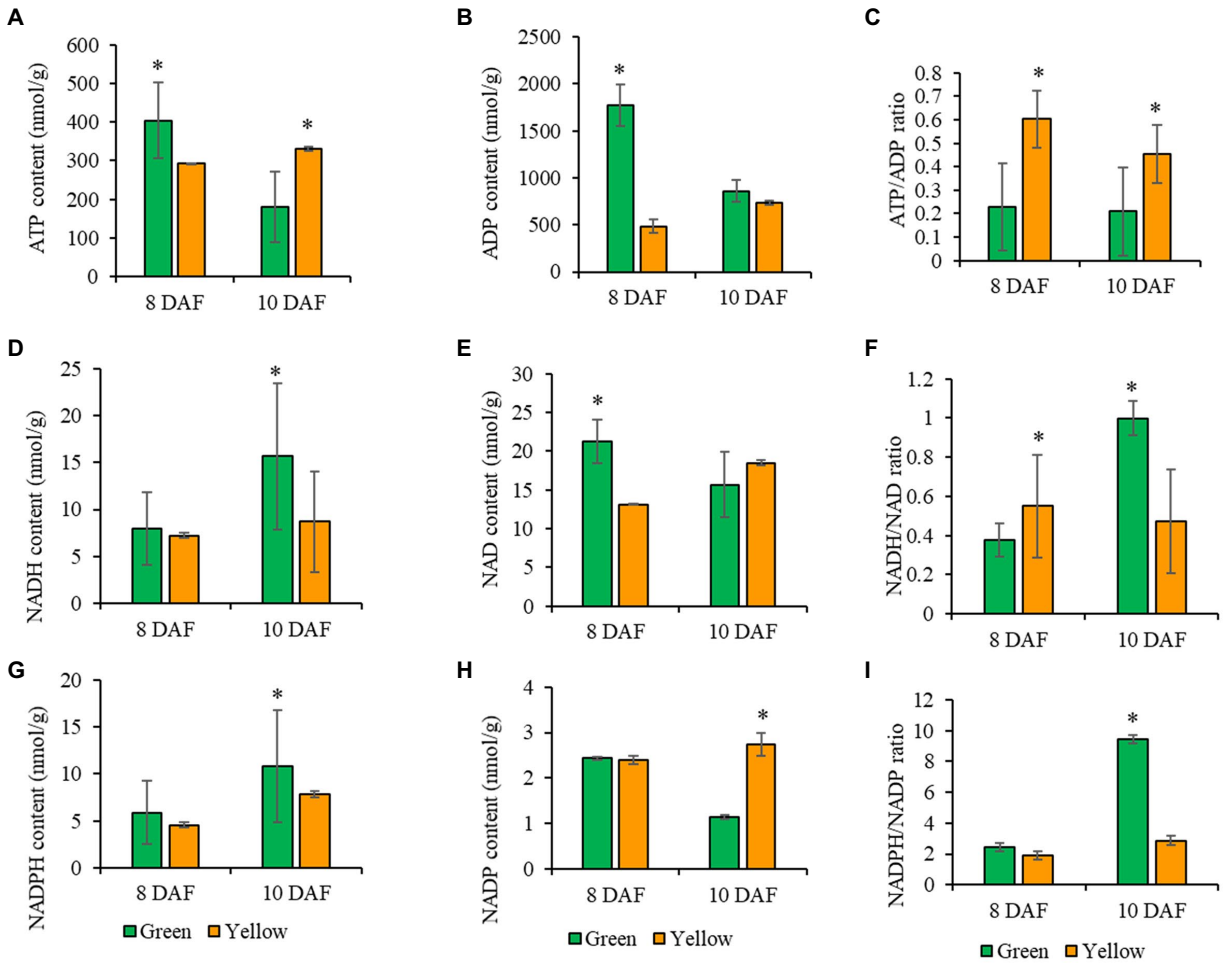
Measurement of pyridine nucleotides and ATP analyses of photosynthetic and non-photosynthetic seeds at 8, and 10 DAF. **(A)** ATP content. **(B)** ADP content. **(C)** ATP/ADP ratio. **(D)** NADH content. **(E)** NAD content. **(F)** NADH/NAD ratio. **(G)** NADPH content. **(H)** NADP content. **(I)** NADPH/NADP ratio. Green bar represents photosynthetic seeds, and the orange bar represents non-photosynthetic seeds. Values are means ± SD (*n* = 3 biological replicates). Statistical significance was analyzed using a two-sided Student *t*-test. The asterisk indicates a significant difference (^*^p < 0.05).

We observed significant changes in ATP levels in photosynthetic, and non-photosynthetic, seeds at 8 DAF (Figure 5A). ADP concentrations in non-photosynthetic seeds were significantly lower compared to ADP levels in photosynthetic seeds (Figure 5B) but were 2-fold higher than ATP. At 10 DAF, ATP levels in non-photosynthetic, seeds change little from concentrations at previous time points, but ATP pool size decreased significantly in photosynthetic seeds. At the same time, ADP concentrations in non-photosynthetic seeds were significantly lower compared to ADP concentrations in photosynthetic, green seeds which were 1.7-fold higher than ATP. The changes observed in ADP concentrations (Figures 5B,C) resulted in a higher ratio of ATP in non-photosynthetic seeds, when compared to control (Figure 5C). Here, the 2-fold increase in the ATP/ADP ratio in non-photosynthetic seeds indicates that these seeds utilized less energy than the photosynthetic green seed. This result seems consistent with the reduction in seed biomass and protein contents in non-photosynthetic seeds (Figures 1, 4). Because ATP is an essential cofactor that drives various cellular biochemical processes in living organisms (Bonora et al., 2012), it seems that inhibition of photosynthesis in non-photosynthetic seeds probably abolishes ATP- and reductant-dependent cellular metabolism, which could have limited the ATP consumption by the cellular metabolism, resulting in accumulation of ATP or an increased ATP/ADP ratio.

Substrate level phosphorylation in the plastid or mitochondria could impact energy production (ATP) through NADH generation (Agrawal and Calvin, 1971; Alberts et al., 2002; Voon et al., 2018). In this study, the NADH levels at 8 DAF in photosynthetic and non-photosynthetic seeds were not significantly different but was slightly reduced in non-photosynthetic seeds (Figure 5D). At 10 DAF, NADH levels was significantly lower in non-photosynthetic seeds compared to photosynthetic seeds (Figure 5D). The NAD concentrations at 8 and 10 DAF in photosynthetic and non-photosynthetic seeds were not significantly different, but consistently reduced in non-photosynthetic, seeds, which resulted in a low NADH/NAD ratio in both seeds at the time points (Figures 5E,F). In photosynthetic seeds, the NADH/NAD ratio increased between 8 and 10 DAF. This increase may be supported by photosynthesis and is important for developing photosynthetically active embryos or transition from the globular to heart stage embryos, during which chloroplast development is enhanced and perhaps required for future accumulation of lipids. On the other hand, in the non-photosynthetic seeds, the NADH/NAD ratio did not increase as seen in the photosynthetic seeds. Because of inhibition of seed photosynthesis in the chloroplasts in non-photosynthetic seeds, the cellular redox state could not be maintained as high as in photosynthetic seeds.

Photosynthesis and the oxidative pentose phosphate pathway are the primary source of NADPH in the plastid, and NADPH supply and utilization are closely linked to FA biosynthesis (Agrawal and Calvin, 1971; Randhir, 1998; Alberts et al., 2002; Andriotis et al., 2010; Andriotis and Smitha, 2019; Carey et al., 2020). In this study, the NADPH levels in photosynthetic and non-photosynthetic seeds were comparable at 8 DAF (Figure 5G). The same levels of NADP concentration were also observed in photosynthetic and non-photosynthetic seeds at 8 DAF (Figure 5H). At 10 DAF, NADPH content decreased by almost 2-folds in non-photosynthetic seeds compared to photosynthetic, green seeds. Whereas NADP concentrations increased by over 2-folds opposite to photosynthetic green seeds (Figure 5H). In photosynthetic seeds, the NADPH/NADP ratio increased remarkably between 8 and 10 DAF. This increase is probably due to photosynthetic activity in photosynthetic seeds. Oppositely, in the non-photosynthetic seeds, such increase in NADPH/NADP ratio was totally abolished, indicating significant utilization of reductants for biosynthetic cellular activities (Figures 5G–I). In the present study, it is likely that the OPPP underpinned the NADPH/NADP ratio observed in non-photosynthetic seeds. To conclude, we hypothesize that the OPPP might play a dominant role in the supply of NADPH over seed photosynthesis in Arabidopsis seeds.

### Gene Expression Studies Provide Clues to Other Pathways of Reductant Supply in Seeds

To gain insights into the transcriptional responses to lack of seed photosynthesis and the genes and pathways involved in the supply of carbon and reducing power (NADPH and ATP) at the early stage of seed development, mRNA-sequencing and qPCR-based genes expression studies were conducted with non-photosynthetic and photosynthetic seeds at 10 DAF (Figure 6; Supplementary Figures S5–S8; Supplementary Tables S2–S7; Supporting Results). The photosynthetic seeds were used as control. The results of RNA-Seq data preprocessing and read alignment are reported in (Supplementary Tables S2–S6; Supplementary Figure S5). Significant changes (*p* < 0.01, FDR = 0.05, log2FC > 1) in gene expression was detected among 230 genes between the non-photosynthetic and photosynthetic seeds (Figures 6A,B). About 94% of these genes were up-regulated and 6% were down-regulated in the pairwise comparison of yellow and green seeds transcripts (Y/G) as shown in Figure 6A. Gene ontology (GO) analysis showed that 177 genes were assigned to the biological process category, 171 genes were assigned to molecular functions and 193 genes were assigned to the cellular component organization (Supplementary Figures S6, S7; Figure 6C). The DEGs were broadly classified into cellular processes, environmental information processing, genetic information processing, metabolism and organismal systems of KEGG pathways (Supplementary Figure S7; Figure 6D). The plant-pathogen interaction, flavonoid biosynthesis, protein processing in the endoplasmic reticulum, carotenoid biosynthesis and starch and sucrose metabolism pathways were significantly enriched (*p* < 0.05; Figure 6D; Supplementary Figure S7). The complete result of GO, clusters of orthologous groups (COG) function classification and KEGG Pathway enrichment analysis (FDR < 0.05, *p* < 0.01) are reported in Supplementary Figures S6, S7.

Further analysis of our RNA-seq data focused on the activity of gene transcripts encoding enzymes involved in expenditure and generation of reducing power. For gene functions related to the expenditure of NADPH, RNA-seq data in agreement with qPCR analysis showed that genes involved in TAG biosynthetic (LPP2, LPAT2, ATPAP1, PAP1, GDSL-motif esterase, FTM1, and PLIP1) and elongation (AT2G17845 and AT2G37540 coding for NAD(P)-binding Rossmann-fold superfamily protein, and KCR2) were significantly induced in the non-photosynthetic seeds (Figure 6E; Supplementary Table S5).

A similar trend was observed in the expression of genes coding for proteins involved in polysaccharide and protein synthesis and metabolism. For instance, we found 15 genes involved in xyloglucan biosynthesis significant up-regulation in non-photosynthetic seeds (Supplementary Tables S5 and S6). Similarly, 33 genes involved in nitrogen compound metabolism were positively induced, in addition to several transcription factors (Supplementary Tables S5 and S6). Together, the seed photosynthetic supply of reducing power is necessary for the normal expression of these genes, given their altered expression in the non-photosynthetic seeds. Moreover, biosynthesis of complex polysaccharides from glucose, and the polymerization of amino acids to form proteins comes at the cost of reducing power (Copper and Hausman, 2016; Heldt and Piechulla, 2016). Because these genes mentioned above are not directly related to the making of reducing power, we propose that they are seed photosynthesis responsive genes.

The glycolysis, oxidative pentose phosphate (OPPP) and tricarboxylic acid cycle (TCA) pathways are primarily associated with the generation of reducing power (Rolletschek et al., 2012; Stincone et al., 2015). Although the initial steps of glycolysis are ATP-dependent, the later reactions provide 2 ATP and 2 NADPH. The OPPP usually gives 2 NADPH per glucose unit, and TCA accounts for about 24 ATP and 4 NADH molecules (Mustroph et al., 2007; Kim et al., 2012; Rolletschek et al., 2012; Stincone et al., 2015). Exploring the transcriptional profile of genes functionally related to the glycolytic pathway in the non-photosynthetic seeds revealed phosphofructokinase 3 (PFK3) and Enolase 1 (ENO1; Mustroph et al., 2007; Prabhakar et al., 2010; Kim et al., 2012) were positively induced. We also observed a significant induction of 13 oxidoreductase genes, perhaps involved in either electron cycling, cellular homeostasis, or reduction of other compounds that may be related to the generation of reducing power. Equally, we detected a general increase in the expression level of several genes (G6PDs, 6-PGLs, PGDs, and RPE isoforms) belonging to the oxidative and non-oxidative branch of the Pentose phosphate pathway in the non-photosynthetic seeds compared to photosynthetic, green seeds which were confirmed by qPCR analysis (Figures 6F,G). Also, we confirmed the significant induction of the GTP2 gene (RNA-Seq FC = 3.2), a plastidic glucose-6-phosphate transmembrane transporter linked to the OPPP, was more than 3-folds higher in the non-photosynthetic than the photosynthetic, green seeds (Figure 6F). Furthermore, the analysis of the gene interaction network provided mechanistic clues that supported role of GTP2 (Niewiadomski et al., 2005; Athanasiou et al., 2010; Araújo et al., 2012) and the link to OPPP as major pathway for NADPH supply in non-photosynthetic seeds (Supporting Results; Supplementary Figure S8; Supplementary Table S5). In addition to other induced genes that may play role in the adjustment to carbon (C)- and energy-limiting growth conditions in the yellow seeds such as ELIP1, EXO EXL1 genes (Figure 6H; Hutin et al., 2003; Schroder et al., 2012). In this study, we confirmed that non-photosynthetic seeds were able to coordinate pathways capable of generating reducing power to support primary cellular function (Figures 6D,E). Here, we propose that OPPP might supply the reducing equivalent required to maintain FA biosynthesis in non-photosynthetic seeds, including the provision of ribose 5-phosphate for nucleotides synthesis (Kammerer et al., 1998; Niewiadomski et al., 2005; Copper and Hausman, 2016; Heldt and Piechulla, 2016). However, further studies are required to test these theories at the post-transcriptional and translational level of gene regulation.

## Conclusion

For many decades, the potential contribution of photosynthesis-derived reductant for fatty acid biosynthesis in green seeds have remained unresolved. By using mutants impaired in chlorophyll synthesis and silique shading experiments; coupled with the measurement of seed oils and oil body ultrastructure, our estimates indicate that reductants derive primarily from photosynthesis in silique (40%) and leaf/stem (40%) compared to seed (20%) make the biggest contribution to FA biosynthesis in green seeds. Our interpretation of these findings is that photosynthetic products imported into the seed from the silique and/or leaf/stem are probably the most important source of carbon and reducing power for seed oil production. Our conclusions are in alignment with early studies that suggest that increase supply of carbon substrates may underpin the synthesis of storage compounds in seeds in which photosynthesis is limited (Wakao et al., 2008).

The role of light and seed photosynthesis in FA accumulation in rapeseed, Arabidopsis, and soybean were proposed based on previous measurements of biosynthetic flux and energy balances in growing embryos *in vitro* (Quebedeaux and Chollet, 1975; Fuhrmann et al., 1994; Ruuska et al., 2004; Goffman et al., 2005; Allen et al., 2009; Zhang et al., 2015). However, genetic evidence from the present work and other studies demonstrates that seed photosynthesis may not be the sole source of carbon and reducing power supporting FA biosynthesis and accumulation in Arabidopsis seeds (Borisjuk et al., 2005; Rolletschek et al., 2005; Wakao et al., 2008; Borisjuk and Rolletschek, 2009) but makes a modest contribution which is important especially at the early stage of seed development (Figures 2–4). Here, we have confirmed an inverse relationship between seed photosynthesis and oil biosynthesis/accumulation (Figure 2A), and the pyridine nucleotides and ATP analyses show that without photosynthesis cellular NAD(P)H/NAD(P) levels cannot be maintained high enough to support FA and lipid synthesis at the early stage of seed development (Figure 6I). Nevertheless, we speculate that oxidative pentose phosphate pathway (OPPP) supplied reductant for *de novo* fatty acid biosynthesis in non-green mutant seeds used in this study (Figures 1–3, 6) which is in agreement with preliminary observations from the characterization of PGL5 (cytoplasmic) and PGL3 (Chloroplastic) mutant seeds (Unpublished data).

Also, our transcriptomic studies implied increased carbon import into the non-photosynthetic seed plastid possibly from other photosynthetic tissues, as seen in the significant up-regulation of key genes of polysaccharide metabolism, and interactions with other pathways that could support the overall growth and development of non-photosynthetic embryo particularly genes involved in cellular homeostasis and adjustment to carbon and energy limiting condition, i.e., ELIP1, EXO and EXL1 (Figure 5H). Here, we uncovered another dimension in the transcriptional response to the general lack of chlorophyll in these seeds early in development, which involved coordinated action of several transcription factors, induction of many heat stress response genes, in addition to redox-stress responsive genes and carbohydrate catabolism, and sugar transport (Figure 5; Supporting Results; Supplementary Figure S8; Supplementary Table S7). For example, we observed the positive induction of GBSS1, APL4, DPE2 genes including GTP2 in the RNA-Seq data and qRT-PCR revealed the up-regulation of several OPPP enzymes in the non-photosynthetic seeds (see Supporting Results; Supplementary Figure S8; Figure 5). Therefore, it is likely that up-regulation of genes primarily involved in carbohydrate biosynthesis, metabolism and transport supported metabolism and translocation of carbon sources needed for *de novo* FA biosynthesis in the yellow seed. However, more experiment is needed to verify the enzyme activity of OPPP genes to further confirm their role in supply of reducing power in green developing seeds.

**Figure 7:**
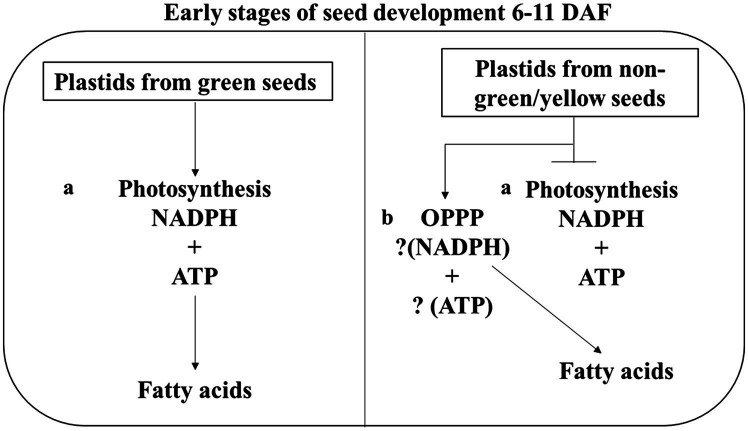
Proposed model of reductant supply for FA biosynthesis seed. This model shows that green developing seeds have two pathways for generating NADPH for FA synthesis during seed development. i.e., **(A)** Photosynthesis **(B)** Oxidative pentose phosphate pathway (OPPP). Six to eleven days after flowering were chosen because seeds de-greening starts at 11 DAF.

Nevertheless, taken all our results together, we propose a model for the supply of reducing power for FA biosynthesis in developing green seeds at the early stages of development (Figure 7). In this model, imported carbon could account for the reductant supply in non-green seeds that accumulated ~80% of wildtype oil (Figure 1). As shown in Figure 3, the few oil bodies observed in the non-photosynthetic seeds at the early developmental stage may be due to lack of seed photosynthesis, or that the lack of photosynthesis abolished the carbon fixation and, therefore, carbon supply from translocation could be the sole source of carbon and energy. Glycolysis and OPPP are important for the utilization of imported carbons for ATP and reductant generation, and in non-photosynthetic seeds, the production of reductants seems limited compared with photosynthetic seeds, it is likely therefore the available reductants were selectively utilized for the maintenance of basic metabolism supporting development. Thus, cellular reductants could be not efficiently used for FA biosynthesis, resulting in decreased lipid deposition in 8–10 DAF embryos.

Finally, by exploring the chlorophyll synthase gene in Arabidopsis, our study provides the first genetic framework for the genetic investigation of sources of reducing power required for *de novo* FA biosynthesis in agriculturally important crops. Similarly, our findings lay the foundation for further studies on the specific role of OPPP in different cellular compartments and how they contribute reducing power for seed oil production in green seeds throughout development. This knowledge can be useful in guiding future metabolic engineering opportunities for oilseed crops either through the overproduction of NADPH through Photosynthesis and OPPP dedicated to FA biosynthesis in seeds.

## Data Availability Statement

The original contributions presented in the study are publicly available. This data can be found here: Gene Expression Omnibus, GSE152614.

## Author Contributions

CN, DL, EC, and CZ designed the research. CN, DL, PQ, LL, WZ, YuZ, JX, YY, JC, LH, and FX performed the research. CN, LL, CL, and LG analyzed the data. LG, YoZ, EC, and CZ provided research resources and contributed to research conception. CN, EC, and CZ wrote the manuscript. All authors contributed to the article and approved the submitted version.

## Funding

This project was supported by grants from the National Natural Science Foundation of China (grant no. 31671723) and the China Agriculture Research System (CARS-12) to CZ and National Key Research and Development Plan of China (2017YFE0104800) to YoZ. EC was supported by Hatch Multistate funding from the Nebraska Agricultural Experiment Station.

## Conflict of Interest

The authors declare that the research was conducted in the absence of any commercial or financial relationships that could be construed as a potential conflict of interest.

## Publisher’s Note

All claims expressed in this article are solely those of the authors and do not necessarily represent those of their affiliated organizations, or those of the publisher, the editors and the reviewers. Any product that may be evaluated in this article, or claim that may be made by its manufacturer, is not guaranteed or endorsed by the publisher.
