# Supplementary material for: Genetic and Biochemical Investigation of Seed Fatty Acid Accumulation in Arabidopsis
Source: Front Plant Sci. 2022 Jul 6;13:942054. doi: 10.3389/fpls.2022.942054 (PMC9328158; doi:10.3389/fpls.2022.942054)
Supplement: Supplementary file 1 [file Table_1.DOCX]

Supplementary Material

# Supplementary Data

- 1. mRNA Sequencing data analysis

After sequencing, the reads were aligned onto the Arabidopsis reference genome assembly (TAIR10). The result of the quality assessment of the sequenced data is reported in (Figures S5, S6 and S7, Tables S2, S3, S4 and S5). The analysis of Pearson correlation coefficients amongst biological replicates (R > 0.98, Figures.S7) confirmed the reproducibility of our RNA-seq experiment. The results of sequence mapping indicate a very high concordant pair alignment rate (>95%).

## Gene ontology (GO) classification and enrichment analysis

Gene ontology (GO) analysis showed a total of 177, 171, and 193 genes were assigned to the biological process, molecular functions, and cellular component organization categories, respectively (Figure.S8). The result of gene enrichment test showed many enriched biological processes, of which “response to abiotic stimulus” including genes that respond to heat stress, UV, UV-B radiation, osmotic, and hypoxia were among the top significantly enriched. In addition to “response to chemical stimulus,” which covers genes that respond to chemical acid such as hormones, organic, and inorganic compounds including karrikin. Equally, enrich were genes related to biotic stress response and those associated with the cellular response to nutrient and energy levels (Table S6). Similarly, genes involved in the “polysaccharide metabolic process,” including those of hemicellulose metabolic process, carbohydrate metabolic process such as xyloglucan metabolic process, and cellular glucan metabolism, were also over-represented. Furthermore, the flavonoid biosynthetic and metabolic process, phosphor-relay signal transduction system, single-organism process, defense process, and innate immunity were enriched. The GO terms of “binding,” “catalytic activity,” and “transporter activity” are the main groups of enriched molecular function category (Table S6). The GO terms “cell wall,” “cell periphery,” “apoplast,” “extracellular region” and “external encapsulating structure” were the most common categories in the cellular component (Table S6).

## COG and KEGG enrichment analysis

The DEPs of Y vs. G comparison were distributed across 17 COG categories, respectively (Figures. S7). Furthermore, the COG analysis revealed significantly differentially regulated gene functions in the Y VS. G comparisons (Figure.S7a). For example, the most abundant COG categories for Y vs. G comparison were the genes with General function prediction or unknown (R) followed by carbohydrate transport and metabolism (G). Other abundant COGs were, transcription (K), signal transduction mechanism (T), post-translational modification, protein turnover, chaperones (O), replication, recombination and repair (L), secondary metabolite biosynthesis, transport and catabolism (Q) (Figure S7a).

KEGG pathway classification revealed DEGs grouped into environmental information processing, genetic information processing, metabolism, and organismal systems, respectively (Figures S9, S10, S11). The Plant-pathogen interaction, Flavonoid biosynthesis, Protein processing in the endoplasmic reticulum, Carotenoid biosynthesis, and Starch and sucrose metabolism.

Analysis of the gene interaction network provides clues to the mechanism of alternative pathway of NADPH supply in non-photosynthetic seeds

Gene expression networks are useful in understanding interactions within biological systems. Here topological analysis of the association of DEPs in the non-photosynthetic seed background (Figure S12), identified several genes as hubs (Table S7), including four functional modules within the network that are related to heat stress, transcriptional process, flavonoid synthesis, carbohydrate metabolism and transport (Figure S12).

We reasoned that the lack of seed photosynthesis might directly or indirectly promote the positive interaction of these genes within the network, which could provide clues to the alternative pathway of NADPH supply for seed oil production. For example, the transcript abundance of heat shock proteins (HSPs) was remarkable (11 HSP genes were positively induced), in addition to other co- chaperons WEE1 (FC=1.1), ROF2 (FC=2.3) and At1g66080 (FC=1.1). This interaction seems critical for protecting the non-photosynthetic developing embryo from stress occasioned by the absence of chlorophyll at the early stages of development. Some studies have shown that HSPs protect unfolding protein intermediates and assists the non-covalent folding of proteins in an ATP-dependent manner during stress (Rowlands et al., 2010; Pearl et al., 2016).

Furthermore, the presence of Cytochrome P450 family proteins CYP707A1 (FC=1.5) and CYP707A3 (FC=2.5); two calcium-binding like proteins CML23, (FC=1.4) and TCH2 (FC=1.3); and PFK3 (FC=1.1); and their direct interaction with HSP90.1 (FC=1.1) amplified the role of HSPs in the multi-cellular processes (Saito et al., 2004; Reddy et al., 2011). The high induction of CYP707A3 suggests it played a vital role in the network, and GO analysis revealed that CYP707A3 is a crucial enzyme of oxidative degradation of abscisic acid (Saito et al., 2004). This reaction yields an oxidized form of NADPH and water.

To further consider the interaction of HSPs with CBPs and PFK in the network, we found reports suggesting that CBPs usually are induced as a cellular response to heat shock (Popescu et al., 2007; Reddy et al., 2011; Elnatan et al., 2018). Moreover, Calmodulins (CaMs) and CaM-like proteins (CMLs) are confirmed to interact with HSPs and PFKs as binding targets (Popescu et al., 2007). Given that these proteins are localized to the cytosol, mitochondria, or plastid, their direct interactions seem to promote cooperative ATP hydrolysis required to drive PFK functions in the glycolytic pathway which could be the source of ATP generation in the non-photosynthetic seeds. It has been reported that calcium can replace magnesium as a cofactor in the mitochondrial in conditions were mg+ supply is limited (Popescu et al., 2007; Elnatan et al., 2018). It is plausible that in the non-photosynthetic seeds, PFK3 interaction with HSP90.1, CML23, and TCH2, might activate the cytosolic glycolytic pathway through cooperative ATP hydrolysis (Roje 2006). And ENO1 (FC=1.0) direct interaction with PFK supported the activation glycolytic pathway in the non-photosynthetic seeds and the production of phosphoenolpyruvate in the cytosol, which could be transported to plastids for FA biosynthesis.

The biological meaning of the direct association of ENO1 and MTO3 (FC=1.1) is unclear. But, GO analysis shows that MTO3 belonged to the S-adenosylmethionine synthetase (SAMs) superfamily protein which is universal methyl group donors, also SAMs are involved in decarboxylation and 5′-deoxyadenosine radicals, to produced propylamine and biotin respectively (Roje 2006). Here MTO3 could regulate the activity of genes (Chen et al., 2016) involved in pyruvate decarboxylation and biotin-binding necessary to generate Malonyl-CoA for FA synthesis at the post-transcriptional level (Sauter et al., 2013; Plaxton 1996). Similarly, SAMs are reported to provide substrates for transmethylation reactions associated with polysaccharide biosynthesis (Asha-Kumari 2018), therefore it seems that the MTO3 and GBSS1 (FC=1.5) direct interaction might result in enhancing starch biosynthesis since GBSS1 is the candidate gene for amylose starch biosynthesis (Shen et al., 2002).

Interestingly, the direct interaction of APL4 (FC=1.8), DPE2 (FC=1), and GBSS1, with GPT2 (FC=3.2), supported an efficient mechanism of polysaccharide catabolic process, which could contribute carbon for the generation of reducing power in the non-photosynthetic seeds (Figure. 12). APL4 catalyzes the first and limiting step in starch biosynthesis (Tenorio et al., 2003; Seung et al., 2015; Sulmon et al., 2011), DPE2 hydrolyze starch to glucose (Ventriglia et al., 2008; Malinova and Fettke 2017), and GPT2 transports glucose into the stroma. it is reasonable to suggest that seed development and reductant supply in the non-photosynthetic seeds is reliant on these sets of gene interactions, which ultimately points to the direction of the OPPP, in part due to the action of GTP2 and the qPCR profile of PGLs. To support these hypotheses, we found reports that show that the expression of GPT2 is required for dynamic acclimation of the plant to high-light (Streb and Zeeman 2012; Athanasiou et al., 2010), and GPT2 is solely responsible for the transport of glucose-6-phosphate (G6P) into plastids of heterotrophic tissues (Kammerer et al., 1998; Niewiadomski et al., 2005).

**Reference**

**Asha-Kumari. A.** (2017) Fatty Acid Biosynthesis Sweet Biochemistry. Remembering Structures, Cycles, and Pathways (Mnemonics). Academic Press.

**Athanasiou, K., Dyson, B. C., Webster, R. E., Johnson. G. N.** (2010) Dynamic acclimation of photosynthesis increases plant fitness in changing environments. Plant Physiol. 152 (1), 366-73.

**Chen, Y., Zou, T., McCormick. S.** (2016) S-Adenosylmethionine Synthetase 3 Is Important for Pollen Tube Growth. Plant Physiol. 172(1):244-53.

**Elnatan, D., Agard. D.A.** (2018) Calcium binding to a remote site can replace magnesium as cofactor for mitochondrial Hsp90 (TRAP1) ATPase activity. J Biol Chem. 293(35):13717-13724.

**Kammerer, B., Fischer, K., Hilpert, B., Schubert, S., Gutensohn, M., Weber, A., Flügge. U. I.** (1998) Molecular characterization of a carbon transporter in plastids from heterotrophic tissues: the glucose 6-phosphate/phosphate antiporter. Plant Cell. 10 (1), 105-17.

**Malinova, I., Fettke. J.** (2017) Reduced starch granule number per chloroplast in the dpe2/phs1 mutant is dependent on initiation of starch degradation. PLoS One. 12(11):e0187985.

**Niewiadomski, P., Knappe, S., Geimer, S., Fischer, K., Schulz, B., Unte, U. S., Rosso, M. G., Ache, P., Flügge, U. I., Schneider, A.** (2005) The Arabidopsis plastidic glucose 6-phosphate/phosphate translocator GPT1 is essential for pollen maturation and embryo sac development. Plant Cell. 17, 760-75.

**Pearl. L. H.** (2016). Review: The HSP90 molecular chaperone-an enigmatic ATPase. Biopolymers. 105(8):594-607.

**Plaxton. W. C.** (1996) The organization and regulation of plant glycolysis." Annu Rev Plant Physiol Plant Mol Biol. 47;185-214.

**Popescu, S.C., Popescu, G.V., Bachan, S., Zhang, Z., Seay, M., Gerstein, M., Snyder, M., Dinesh-Kumar. S.P.** (2007) Differential binding of calmodulin-related proteins to their targets revealed through high-density Arabidopsis protein microarrays. Proc. Natl. Acad. Sci. USA, 104:4730-4735.

**Reddy, A.S.N., Ben-Hur, A., Day. I. S.** (2011) Experimental and computational approaches for the study of calmodulin interactions. Phytochemistry. 72(10) 1007-1019.

**Roje S.** (2006) S-Adenosyl-L-methionine: beyond the universal methyl group donor. Phytochemistry. 67:1686–1698.

**Rowlands, M., McAndrew, C., Prodromou, C., Pearl, L., Kalusa, A., Jones, K., Workman, P., Aherne. W.** (2010) Detection of the ATPase activity of the molecular chaperones Hsp90 and Hsp72 using the TranscreenerTM ADP assay kit. J Biomol Screen. 15(3):279-86.

**Saito, S., Hirai, N., Matsumoto, C., Ohigashi, H., Ohta, D., Sakata, K., Mizutani. M.** (2004) Arabidopsis CYP707As encode (+)-abscisic acid 8'-hydroxylase, a key enzyme in the oxidative catabolism of abscisic acid. Plant Physiol. 134(4):1439-49.

**Sauter, M., Moffatt, B., Saechao, M. C., Hell, R., Wirtz M.** (2013) Methionine salvage and S-adenosylmethionine: essential links between sulfur, ethylene and polyamine biosynthesis. Biochem J. 451:145–154.

**Seung, D., Soyk, S., Coiro, M., Maier, B.A., Eicke, S., Zeeman. S.C.** (2015) Protein targeting to starch is required for localizing granule-bound starch synthase to starch granules and for normal amylose synthesis in Arabidopsis. PLoS Biol. 13(2):e1002080.

**Shen, B., Li, C., Tarczynski. M.C.** (2002) High free-methionine and decreased lignin content result from a mutation in the Arabidopsis S-adenosyl-Lmethionine synthetase 3 gene. Plant J. 29:371–380.

**Streb, S., Zeeman. S.C.** (2012). Starch metabolism in Arabidopsis. Arabidopsis Book.10:e0160. doi: 10.1199/tab.0160.

**Sulmon, C., Gouesbet, G., Ramel, F., Cabello-Hurtado, F., Penno, C., Bechtold, N., Couée, I., El Amrani, A.** (2011) Carbon dynamics, development and stress responses in Arabidopsis: involvement of the APL4 subunit of ADP-glucose pyrophosphorylase (starch synthesis). PLoS One. 6(11): e26855.

**Tenorio, G., Orea, A., Romero, J.M., Mérida. A.** (2003). Oscillation of mRNA level and activity of granule-bound starch synthase I in Arabidopsis leaves during the day/night cycle. Plant Mol Biol. 51(6):949-58.

**Ventriglia, T., Kuhn, M.L., Ruiz, M.T., Ribeiro-Pedro, M., Valverde, F., Ballicora, M.A., Preiss, J.,. Romero. J.M.** (2008) Two Arabidopsis ADP-glucose pyrophosphorylase large subunits (APL1 and APL2) are catalytic. Plant Physiol. 148(1):65-76.
