# Supplementary material for: Genetic and Biochemical Investigation of Seed Fatty Acid Accumulation in Arabidopsis
Source: Front Plant Sci. 2022 Jul 6;13:942054. doi: 10.3389/fpls.2022.942054 (PMC9328158; doi:10.3389/fpls.2022.942054)
Supplement: Supplementary file 2 [file Data_Sheet_1.docx]

Supplementary Material

# Supplementary Figures and Tables

## Supplementary Figures

**a**


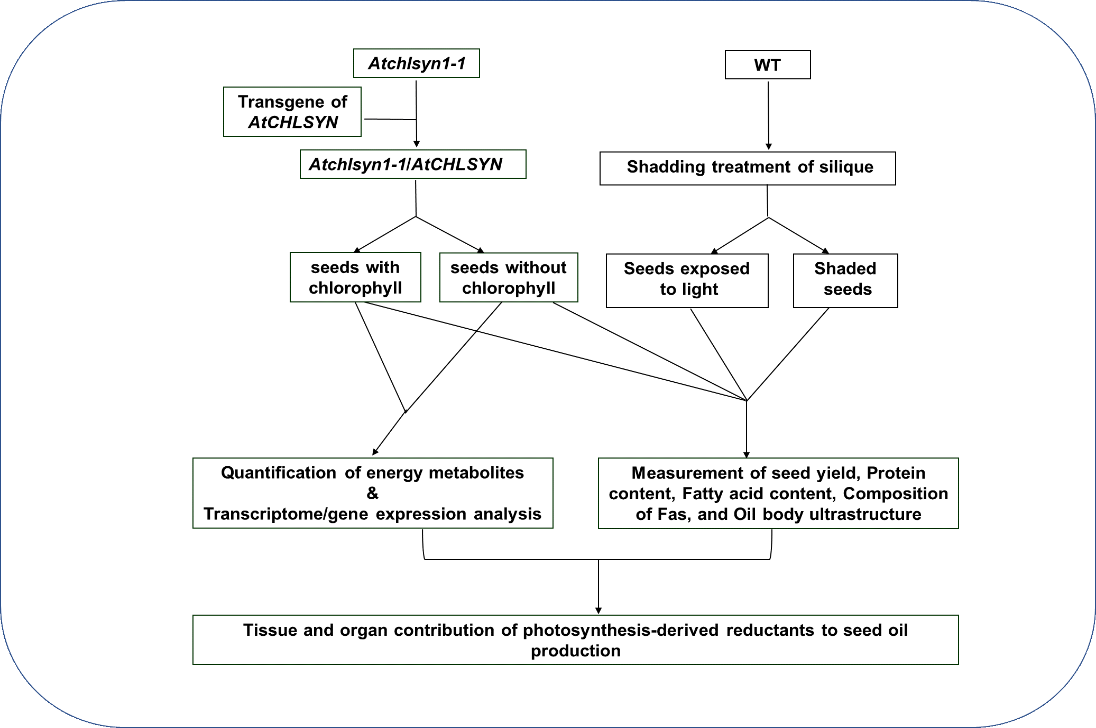


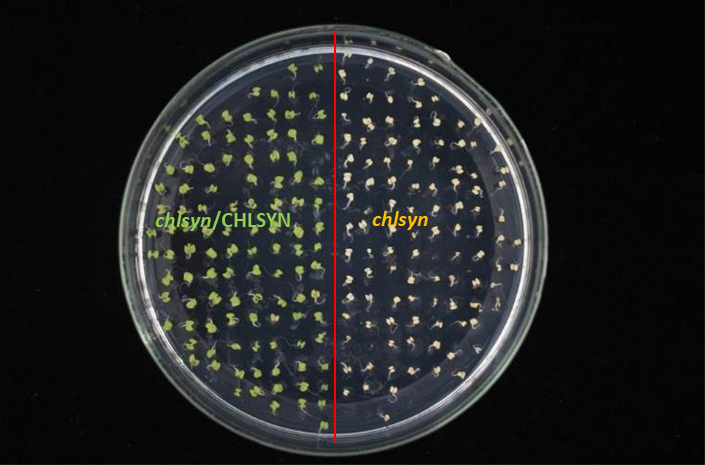


**c**

**c**

**b**

**
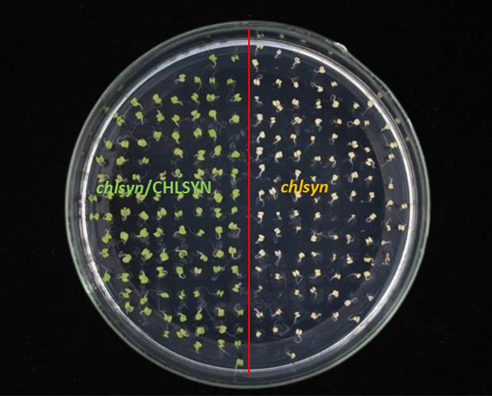
**
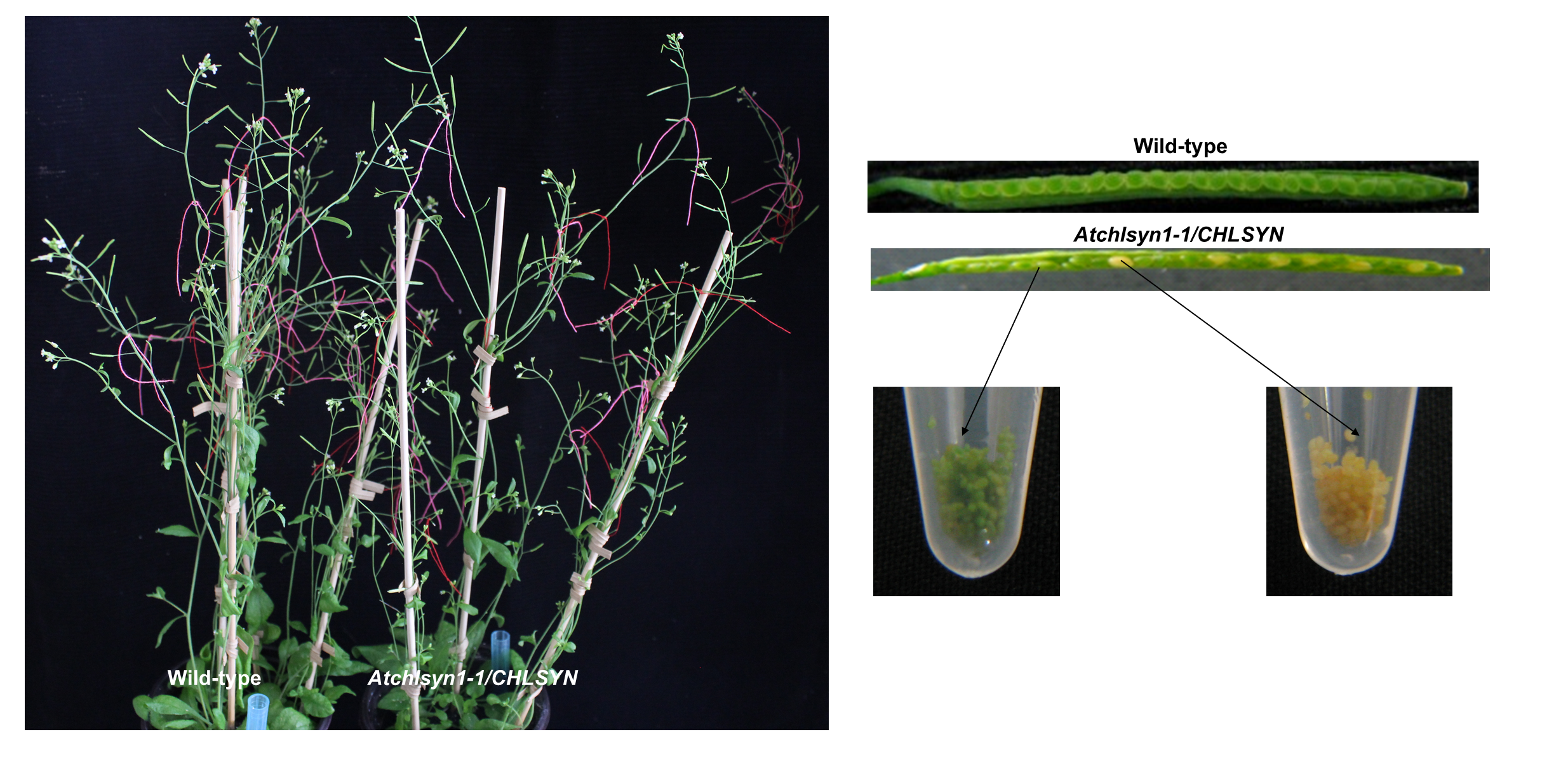


**Supplementary Figure S1.** Experimental overview and analysis of independent Arabidopsis wild-type plant and chlorophyll synthase mutant lines. (a). Flow-chart showing steps used to investigate the sources of reducing power required for fatty acid biosynthesis in Arabidopsis seed. (b). Arabidopsis wild-type plant and Arabidopsis chlorophyll synthase knockout mutant Atchlsyn1-1 complemented with the Arabidopsis chlorophyll synthase gene linked with DsRed marker. (c). Seeds of Arabidopsis chlorophyll synthase knockout mutant Atchlsyn1-1 complemented with the Arabidopsis chlorophyll synthase gene linked with DsRed marker. Heterozygous lines segregate 3-photosynthetic green seeds and 1-non-photosynthetic yellow seed.


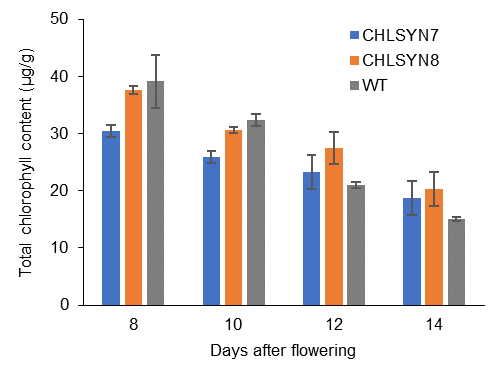


**a**

**Supplementary Figure S2. Analysis of chlorophyll content of WT and CHLSYN mutant green seeds.** The two independent lined WT are represented by blue, orange, and black bars respectively. Values are means ±SD (n = 3 biological replicates). Statistical significance was analyzed using the two-sided Student t-test. The asterisk indicates a significant difference (* p < 0.05).

**
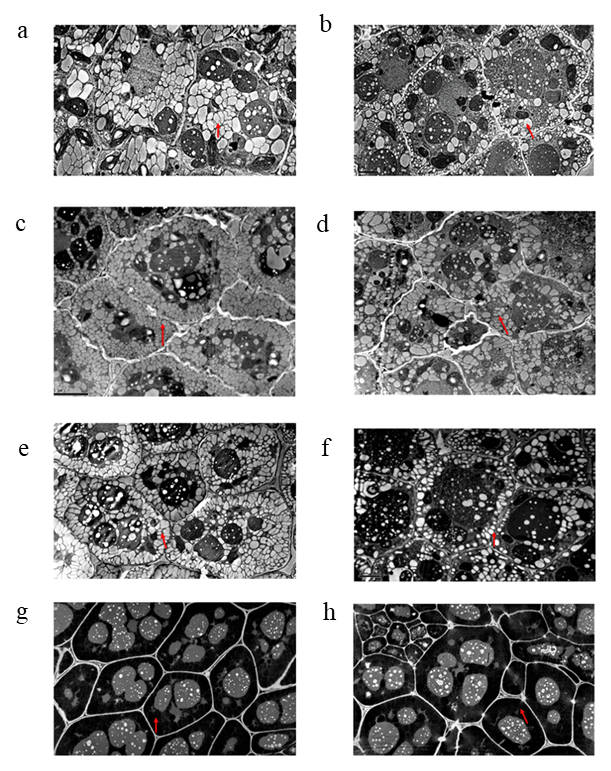
**

**Supplementary Figure S3**. The TEM analysis lipid bodies of embryos from Arabidopsis chlorophyll synthase mutant line CHLSYN7. The TEM analysis of oil bodies of embryos from Arabidopsis chlorophyll synthase mutant line CHLSYN 8. a, c, e, g, represents sections of developing embryos from green seeds at 8, 10, 12 DAF and matured dry seed (Red). b, d, f, h represents sections of developing embryos from yellow seeds at 8, 10, 12 DAF and matured dry seed (Non-red). Red triangles indicate the oil body. Scale bar = 5 μm.


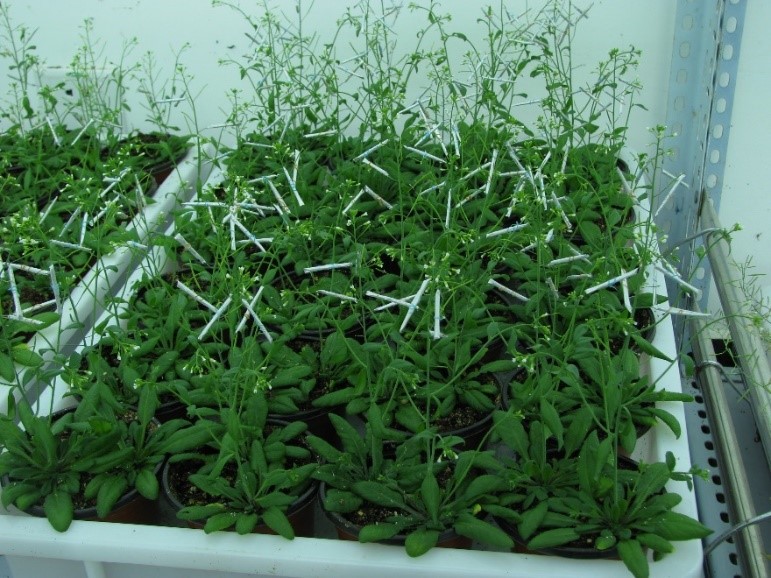


**a**

**Supplementary Figure S4.** Whole life shading experiment of wild-type plants. Shaded siliques were covered with aluminum foil, control plants were unshaded.


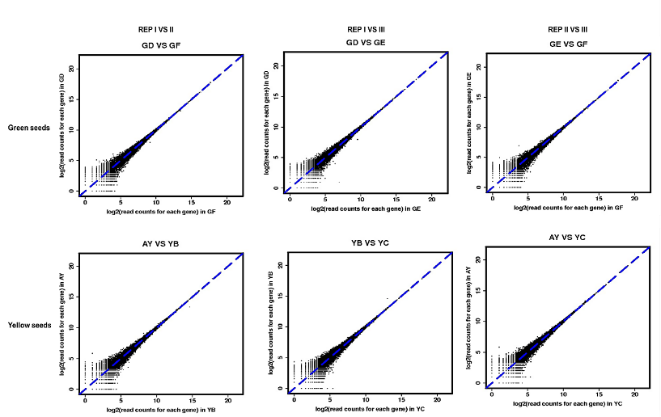

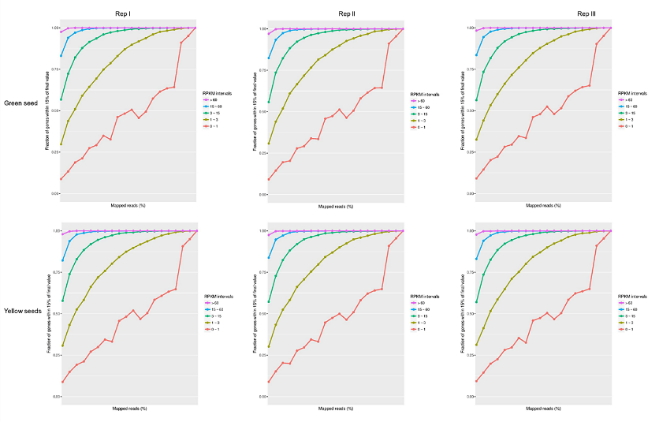


**b**

**a**


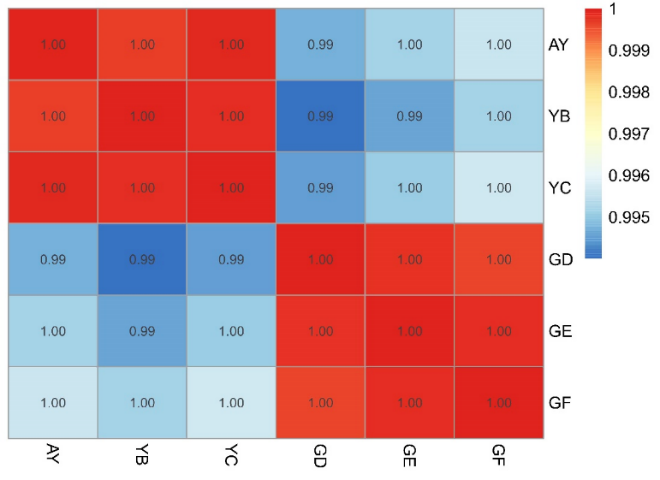


**c**

**Supplementary Figure S5.** **RNA sequencing statistics (a)** The saturation curves for the different samples generated from RNA sequencing data. Each point on the curve was generated by randomly selecting a number of raw reads from each sample library and then call genes with mean FPKM >1. Each point represents three replicate sub-samplings. Error bars, standard error. **(b)** The scatter diagram showing gene expression levels in all samples. Variation in gene expression as a function of gene expression level across sample replicates. **(c)** Pearson correlation analysis of genes in the photosynthetic and non-photosynthetic seeds. Coefficient values within the same color scheme distinguishes the samples.


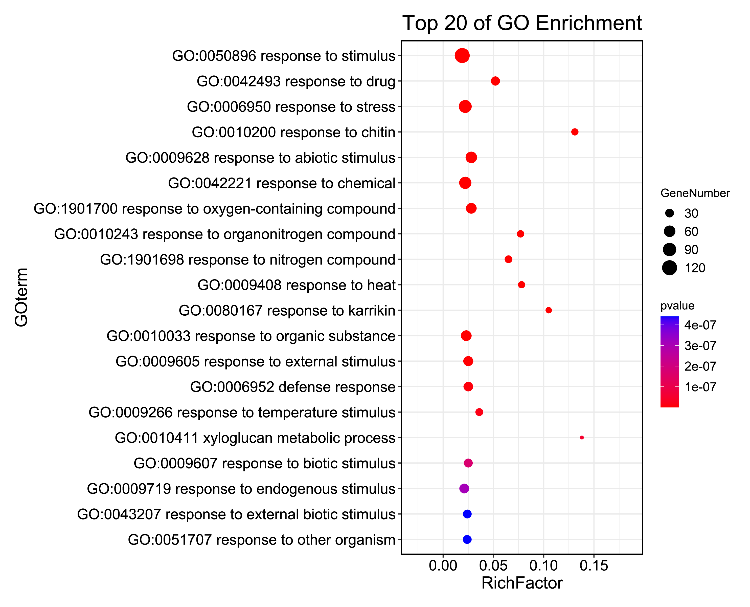

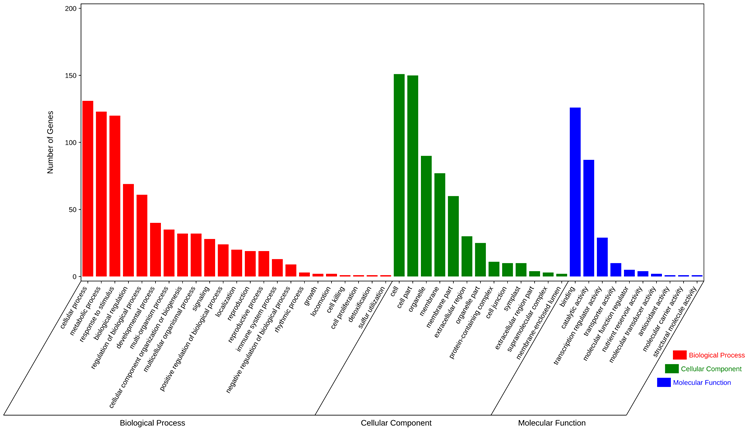


**b**

**a**


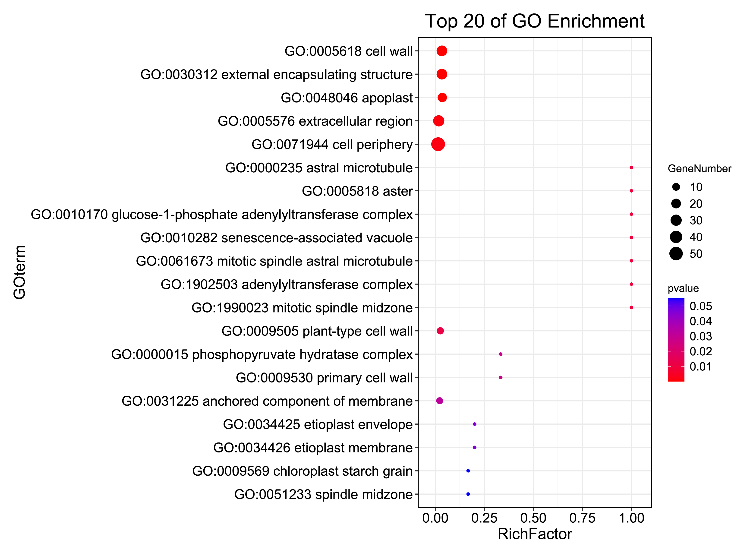

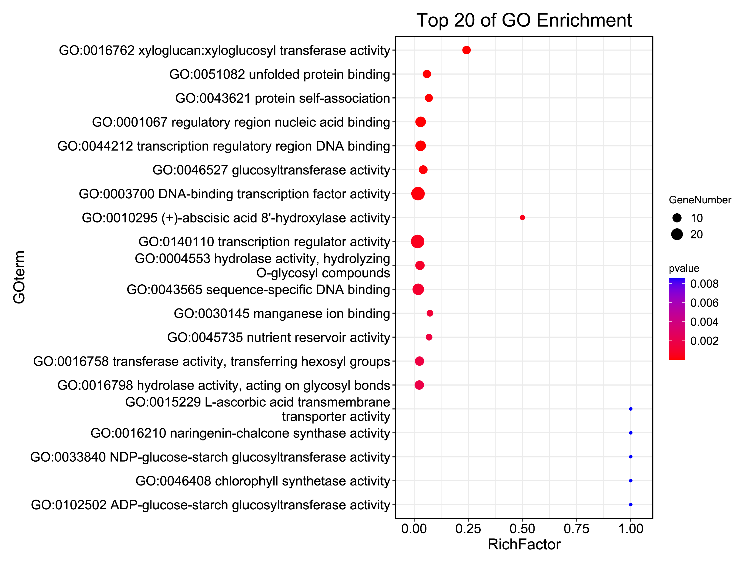


**d**

**c**

**Supplementary Figure S6**. **Gene ontology classification and top enriched GO terms.** (a). GO functional classes, genes are classified into biological process, molecular function and cellular component. (b). Top enriched biological processes, (c). Molecular functions and (d). Cellular component categories. Size and color circle represent number on genes and significant enrichment.


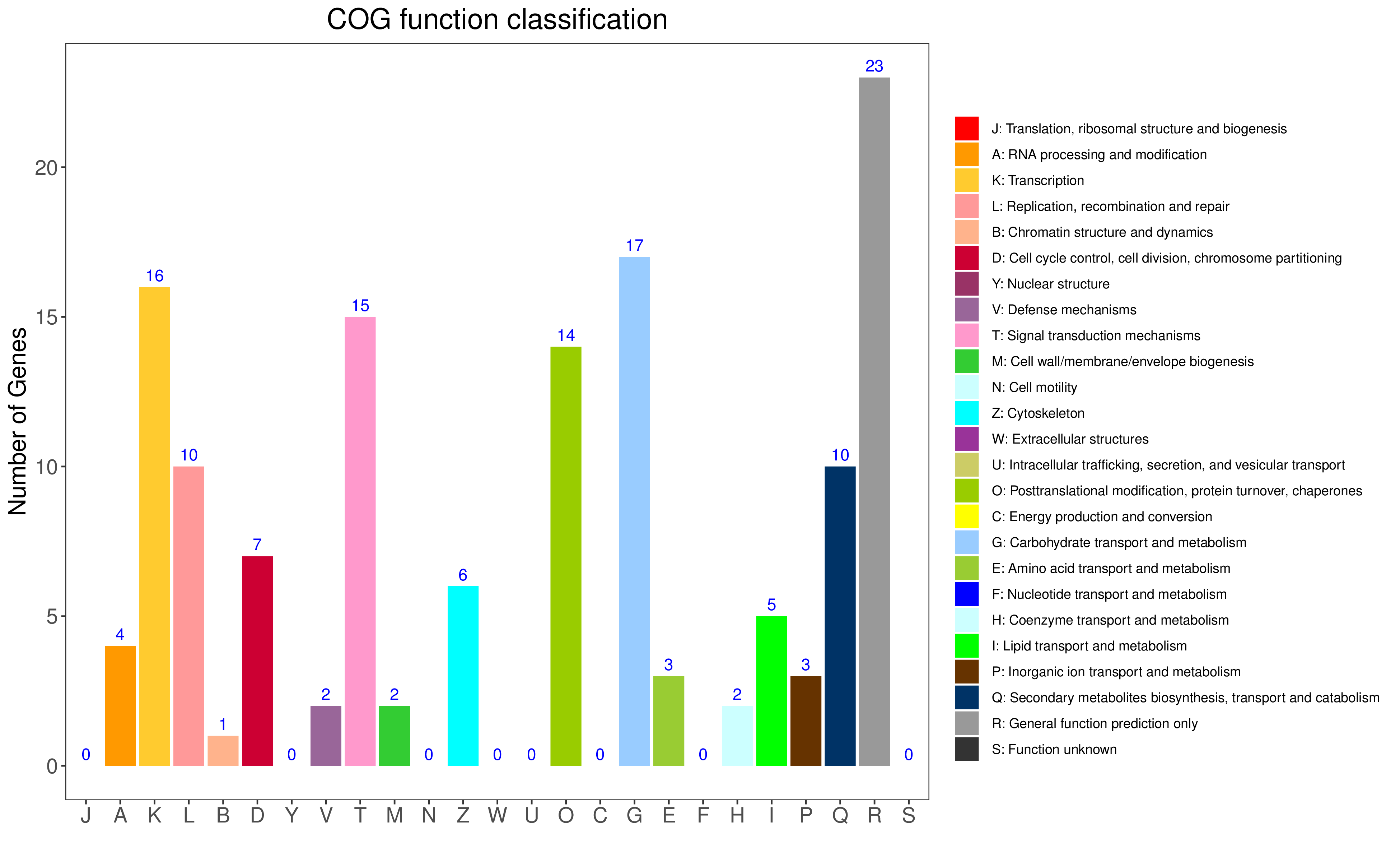

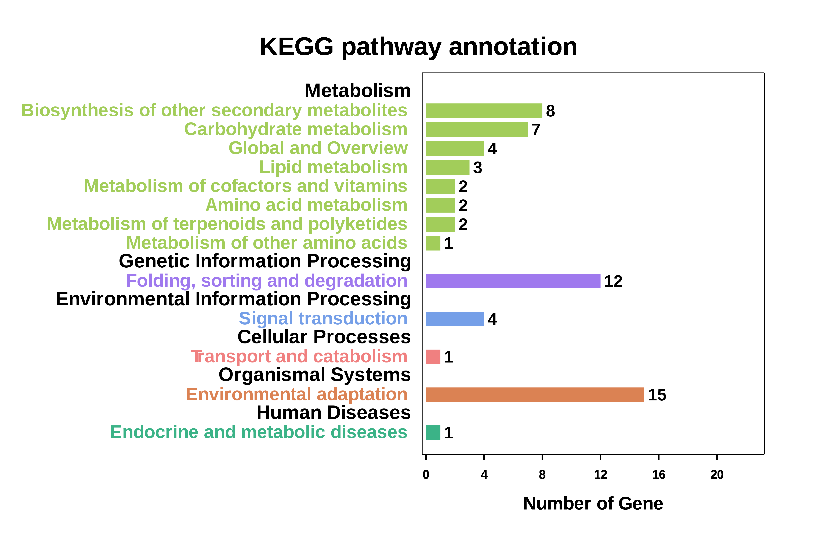


**a**

**b**


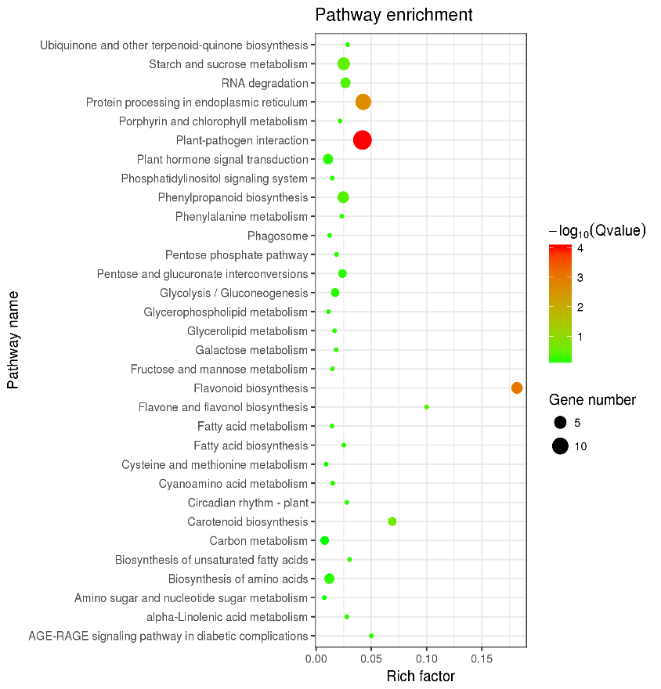


**c**

**Supplementary** **Figure S7.** **(a).** Clusters of Orthologous Groups (COG) Function classification. **(b)**. KEGG Pathway classification of DEPs. **(c)**. KEGG enrichment of DEGs.

**
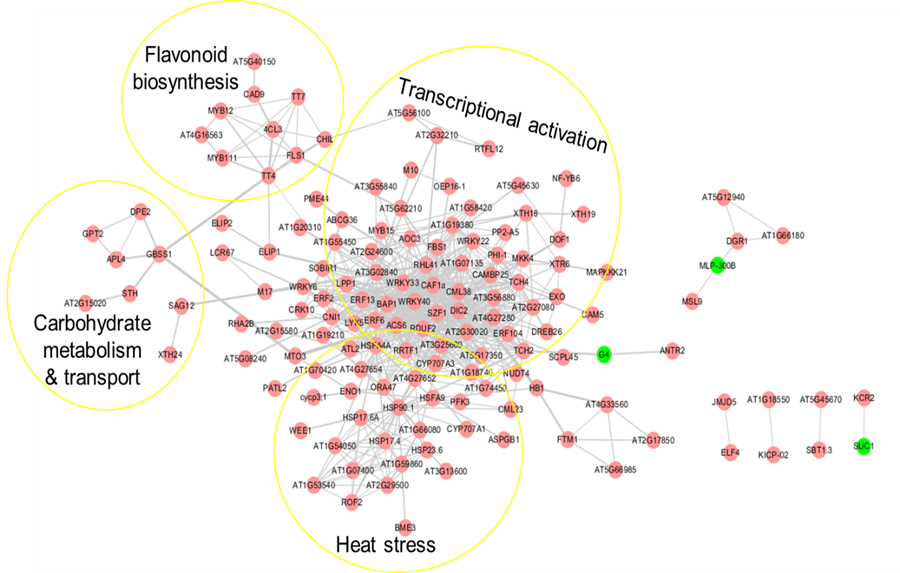
**

**a**

**Supplementary** **Figure S8. Gene interaction networks of response to the lack of photosynthesis identified by RNA-seq experiments**. The yellow circle indicates functional modules within the network such as heat stress, transcriptional process, flavonoid biosynthesis, and carbohydrate biosynthesis, catabolism, and transport. Gray lines represent the co-expression evidence. The beige color indicates up-regulated genes and green color indicates down-regulated genes.

**Supplementary Table S1.** LC/MS/MS data showing standards measurement of ADP, ATP, NADH, NADP, NADPH and NAD

| **Sample Name** | **Component Name** | **Area** | **Retention Time** |
| --- | --- | --- | --- |
| 0.1 | PIPES | 1.99E+07 | 13.28 |
| 0.5 | PIPES | 1.97E+07 | 13.33 |
| 1 | PIPES | 8.09E+07 | 13.28 |
| 5 | PIPES | 1.49E+08 | 13.26 |
| 10 | PIPES | 6.84E+07 | 13.3 |
| 20 | PIPES | 5.60E+07 | 13.31 |
| 50 | PIPES | 5.55E+07 | 13.28 |
| 100 | PIPES | 5.19E+07 | 13.31 |
| Sample Name | Component Name | Area | Retention Time |
| 0.1 | ADP | 8.33E+02 | 37.17 |
| 0.5 | ADP | 1.79E+03 | 37.25 |
| 1 | ADP | 1.83E+03 | 36.99 |
| 5 | ADP | 1.10E+03 | 36.74 |
| 10 | ADP | 3.54E+08 | 37.53 |
| 20 | ADP | 1.17E+09 | 36.92 |
| 50 | ADP | 3.46E+09 | 36.79 |
| 100 | ADP | 5.48E+09 | 36.73 |
| Sample Name | Component Name | Area | Retention Time |
| 0.1 | ATP | 3.06E+02 | 44.19 |
| 0.5 | ATP | 1.99E+03 | 44.18 |
| 1 | ATP | 2.46E+03 | 44.14 |
| 5 | ATP | 4.15E+03 | 44.1 |
| 10 | ATP | 1.99E+08 | 44.53 |
| 20 | ATP | 8.20E+08 | 44.41 |
| 50 | ATP | 2.52E+09 | 44.33 |
| 100 | ATP | 4.28E+09 | 44.26 |
| Sample Name | Component Name | Area | Retention Time |
| 0.1 | NADH | 3.54E+05 | 38.57 |
| 0.5 | NADH | 1.27E+06 | 38.59 |
| 1 | NADH | 4.12E+06 | 38.6 |
| 5 | NADH | 9.07E+07 | 38.54 |
| 10 | NADH | 1.91E+08 | 38.54 |
| 20 | NADH | 4.52E+08 | 38.52 |
| 50 | NADH | 1.03E+09 | 38.48 |
| 100 | NADH | 1.69E+09 | 38.45 |
| Sample Name | Component Name | Area | Retention Time |
| 0.1 | NADP | N/A | N/A |
| 0.5 | NADP | 2.28E+05 | 35.5 |
| 1 | NADP | 9.58E+05 | 35.43 |
| 5 | NADP | 5.90E+06 | 35.18 |
| 10 | NADP | 9.39E+06 | 35.2 |
| 20 | NADP | 2.54E+07 | 35.13 |
| 50 | NADP | 5.00E+07 | 35.09 |
| 100 | NADP | 8.30E+07 | 34.97 |
| Sample Name | Component Name | Area | Retention Time |
| 0.1 | NADPH | 3.47E+02 | 44.5 |
| 0.5 | NADPH | 2.16E+02 | 44.43 |
| 1 | NADPH | 3.98E+02 | 44.34 |
| 5 | NADPH | 1.43E+08 | 44.78 |
| 10 | NADPH | 2.88E+08 | 44.73 |
| 20 | NADPH | 5.91E+08 | 44.71 |
| 50 | NADPH | 1.17E+09 | 44.66 |
| 100 | NADPH | 1.64E+09 | 44.63 |
| Sample Name | Component Name | Area | Retention Time |
| 0.1 | NAD | 6.55E+04 | 23.43 |
| 0.5 | NAD | 2.18E+05 | 23.4 |
| 1 | NAD | 8.76E+05 | 23.28 |
| 5 | NAD | 4.34E+06 | 23.23 |
| 10 | NAD | 7.02E+06 | 23.32 |
| 20 | NAD | 1.28E+07 | 23.31 |
| 50 | NAD | 3.12E+07 | 23.32 |
| 100 | NAD | 5.34E+07 | 23.3 |

**Supplementary Table S2.** Statistics of short-read alignment and mapping of all sequenced reads

| Sample name | Totalpairs | Aligned 0time | Aligned concordantly1time | Aligned concordantly>1 times | Aligned discordantly1 time | unpaired reads | Aligned0time Aligned1time Aligned>1times | Overall alignment rate |
| --- | --- | --- | --- | --- | --- | --- | --- | --- |
| GD | 19901481 | 1710289 (8.59%) | 17076435(85.80%) | 588760(2.96%) | 525997(2.64%) | 3420578 1771728(51.80%) | 1511776(44.20%) | 137074(4.01%) 95.55% |
| GE | 19791397 | 1516602 (7.66%) | 17292874(87.38%) | 565407(2.86%) | 416514(2.10%) | 3033204 1574393(51.91%) | 1344201(44.32%) | 114610(3.78%) 96.02% |
| GF | 19733571 | 1357710 (6.88%) | 17358140(87.96%) | 574841(2.91%) | 442880(2.24%) | 2715420 1393731(51.33%) | 1218209(44.86%) | 103480(3.81%) 96.47% |
| YA | 20487270 | 1903254 (9.29%) | 17400356(84.93%) | 585598(2.86%) | 598062(2.92%) | 3806508 2091175(54.94%) | 1570485(41.26%) | 144848(3.81%) 94.90% |
| YB | 20246765 | 1808146 (8.93%) | 17279316(85.34%) | 658306(3.25%) | 500997(2.47%) | 3616292 1959277(54.18%) | 1512024(41.81%) | 144991(4.01%) 95.16% |
| YC | 19944667 | 1636831 (8.21%) | 17244233(86.46%) | 581769(2.92%) | 481834(2.42%) | 3273662 1772564(54.15%) | 1380346(42.17%) | 120752(3.69%) 95.56% |

**Supplementary Table S3.** Result of quality control on sequenced reads

| **Sample name** | **Raw reads** | **Cleans reads** | **Clean base (bp)** | **Effective (%)** | **Q20 (%)** | **Q30 (%)** | **GC (%)** |
| --- | --- | --- | --- | --- | --- | --- | --- |
| GD | 23694028 | 19901481 | 5919724986 | 83.99 | 9.39;98.19 | 97.61;93.32 | 48.46;48.44 |
| GE | 23632777 | 19791397 | 5890013276 | 83.75 | 99.32;98.42 | 97.42;94.15 | 8.55;48.53 |
| GF | 23576313 | 19733571 | 5868879646 | 83.7 | 99.38;98.23 | 97.57;93.48 | 48.45;48.42 |
| YA | 23547824 | 20487270 | 6104512402 | 87 | 99.42;98.65 | 97.62;94.89 | 48.28;48.26 |
| YB | 24030372 | 20246765 | 6023036013 | 84.25 | 99.37;98.34 | 97.54;93.92 | 48.30;48.28 |
| YC | 23692518 | 19944667 | 5932792302 | 84.18 | 99.36;98.29 | 97.52;93.67 | 48.32;48.30 |

**Supplementary Table S4.** Result of quality control on sequenced reads

| RPKM Interval | 0-1 | 1-3 | 3-15 | 15-60 | >60 | Total |
| --- | --- | --- | --- | --- | --- | --- |
| GD | 16942 | 3198 | 7916 | 3633 | 989 | 32678 |
| GE | 16949 | 3269 | 7806 | 3659 | 995 | 32678 |
| GF | 16976 | 3290 | 7861 | 3574 | 977 | 32678 |
| YA | 17163 | 3376 | 7730 | 3475 | 934 | 32678 |
| YB | 17257 | 3356 | 7766 | 3367 | 932 | 32678 |
| YC | 17185 | 3346 | 7772 | 3440 | 935 | 32678 |

**Supplementary Table S5**. List of genes acting as hubs in the gene expression network

| **Gene ID** | **Description** | **Log2FC** | **Number of connections in the network** |
| --- | --- | --- | --- |
| AT1G80840 | WRKY DNA-binding protein 40 | 1.49 | 46 |
| AT4G24570 | dicarboxylate carrier 2 | 1.55 | 44 |
| AT3G55980 | salt-inducible zinc finger 1 | 1.76 | 41 |
| AT2G38470 | WRKY DNA-binding protein 33 | 1.99 | 40 |
| AT3G44260 | Polynucleotidyl transferase, ribonuclease H-like superfamily protein | 1.18 | 36 |
| AT1G76650 | calmodulin-like 38 | 1.36 | 36 |
| AT4G11280 | 1-aminocyclopropane-1-carboxylic acid (acc) synthase 6 | 1.23 | 34 |
| AT5G59550 | zinc finger (C3HC4-type RING finger) family protein | 1.01 | 33 |
| AT4G27280 | Calcium-binding EF-hand family protein | 1.15 | 31 |
| AT3G02840 | ARM repeat superfamily protein | 1.16 | 30 |
| AT5G59820 | C2H2-type zinc finger family protein | 1.07 | 28 |
| AT4G34410 | redox responsive transcription factor 1 | 1.52 | 25 |
| AT5G27420 | carbon/nitrogen insensitive 1 | 2.16 | 22 |
| AT5G52640 | heat shock protein 90.1 | 1.05 | 19 |
| AT5G45340 | cytochrome P450, family 707, subfamily A, polypeptide 3 | 2.49 | 18 |
| AT5G08640 | flavanol synthase 1 | 1.68 | 7 |
